# Supplementary material for: Well-defined nanostructuring with designable anodic aluminum oxide template
Source: Nat Commun. 2022 May 4;13:2435. doi: 10.1038/s41467-022-30137-6 (PMC9068917; doi:10.1038/s41467-022-30137-6)
Supplement: Supplementary file 1 — Supplementary Information [file 41467_2022_30137_MOESM1_ESM.pdf]

Supplementary Materials for

Well-defined nanostructuring with designable anodic aluminium oxide template

Rui Xu[1], Zhiqiang Zeng[1], Yong Lei[1]*

[1]Fachgebiet Angewandte Nanophysik, Institut für Physik & IMN MacroNano, Technische Universität Ilmenau, Ilmenau 98693, Germany.

Correspondence to: yong.lei@tu-ilmenau.de

This PDF file includes:

Supplementary Figs. 1 to 41

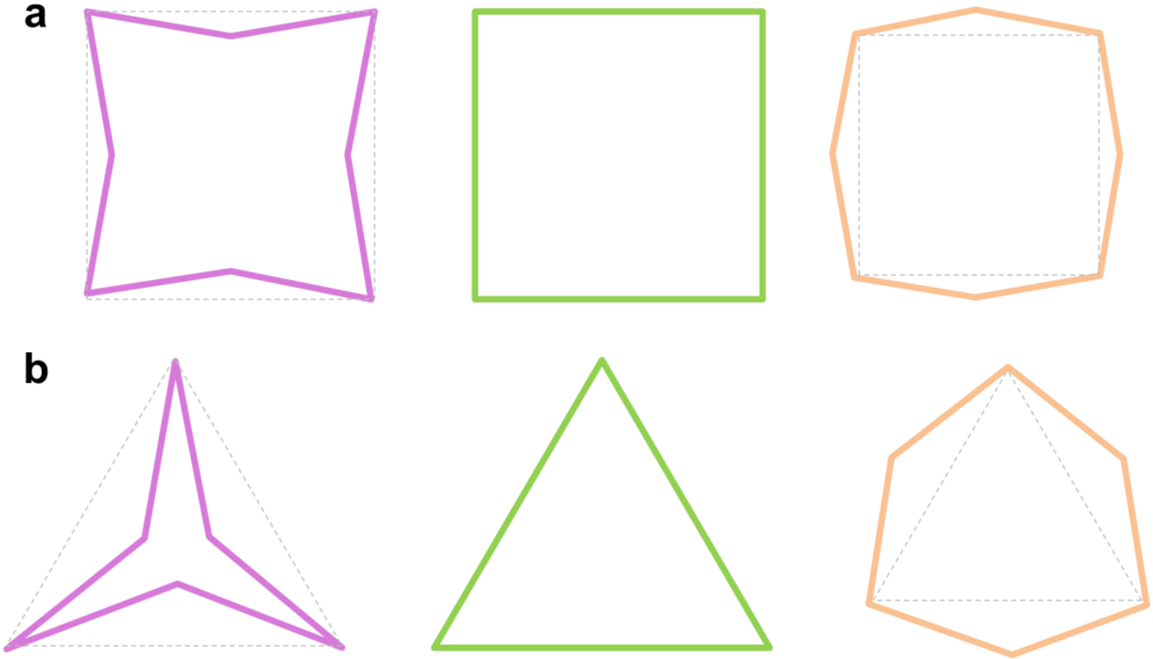

Supplementary Fig. 1 | Schematic illustration of in-plane shape-different pores. (**a**) Square and (**b**) triangle with (from left to right) internally-bent walls, non-bent walls and externally-bent walls.

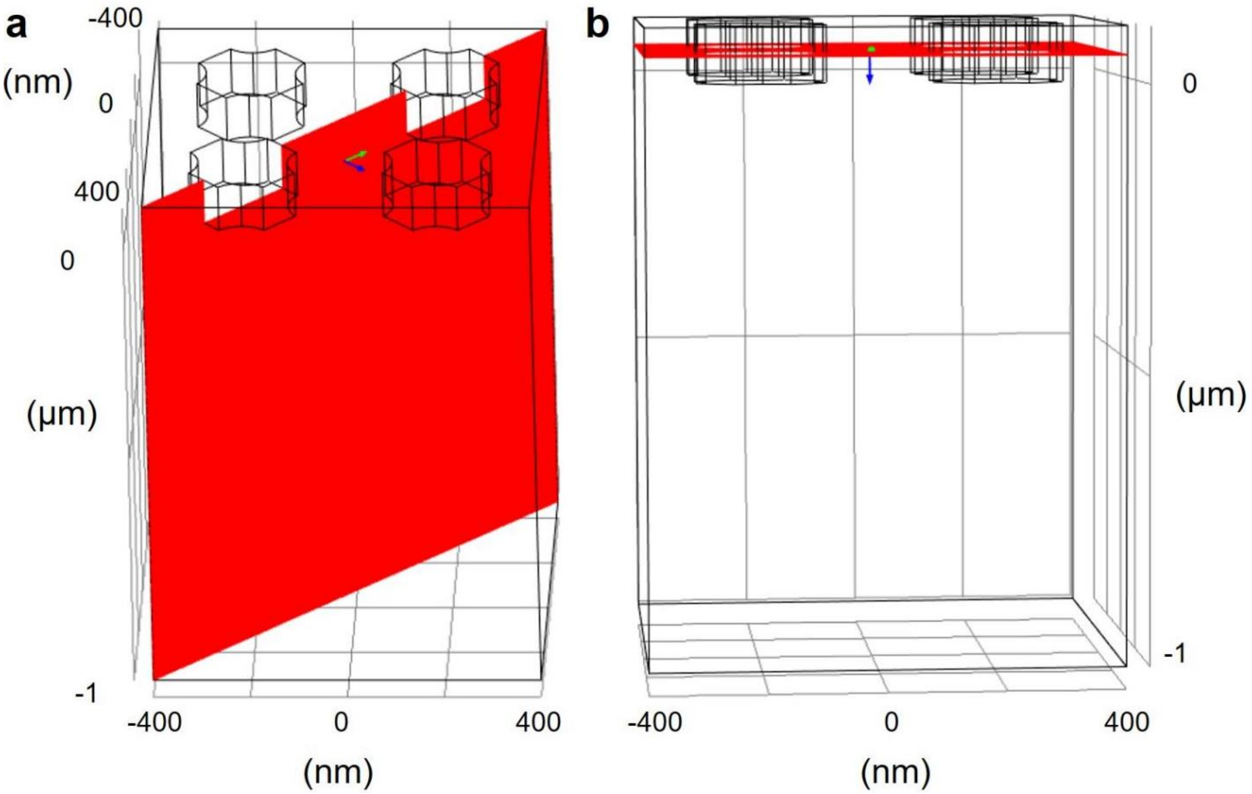

Supplementary Fig. 2 | Three-dimensional layout of surface-patterned aluminium foil in COMSOL simulation. (**a**) Vertical cross section and (**b**) near-surface lateral cross section, cutting through the nanoconcaves in an aluminium foil.

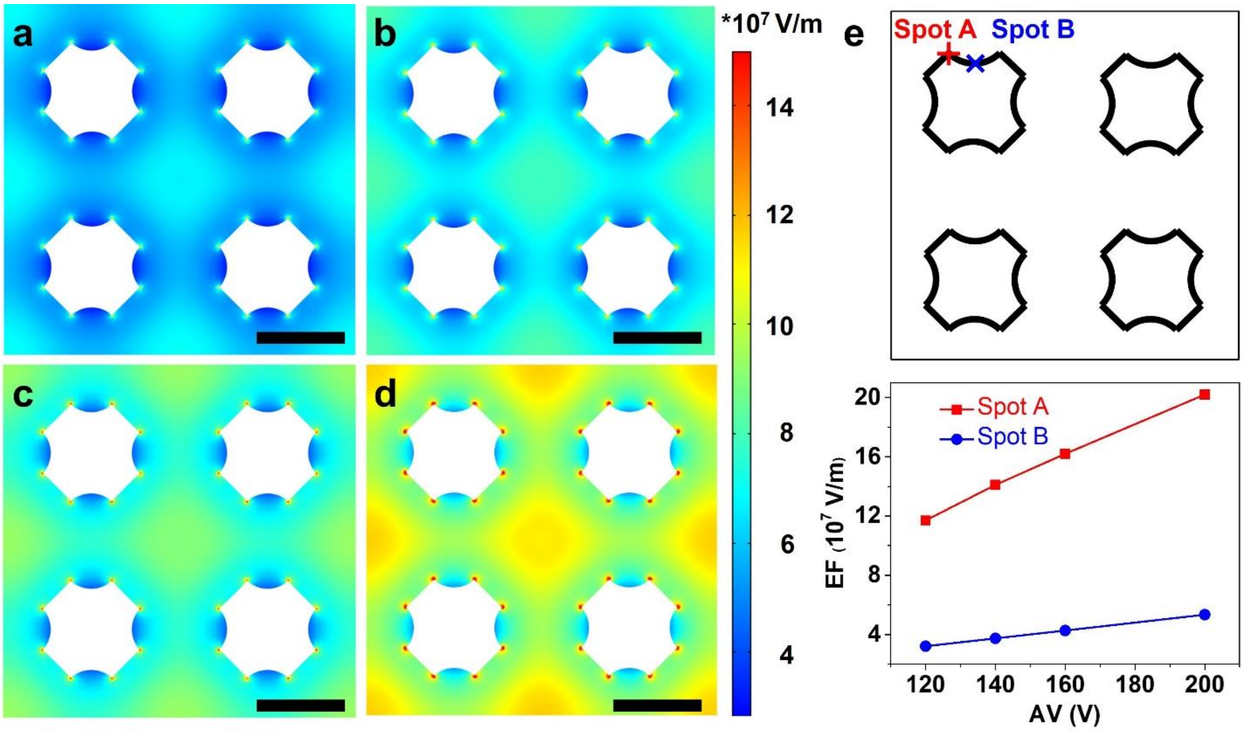

Supplementary Fig. 3 | COMSOL-simulated electric field maps over the near-surface lateral cross section of surface-patterned aluminium foil at different AVs. Electric field maps at the AVs of (**a**) 120 V, (**b**) 140 V, (**c**) 160 V, and (**d**) 200 V. For a clear comparison, the color bars of all electric field maps are set to be identical. The electric field map in (**a**) is derived from (and same as) that in Fig. 1b. Scale bars: 200 nm. (**e**) Schematic illustration of 2 × 2 nanoconcave array and the electric fields at spot A and spot B as a function of AV.

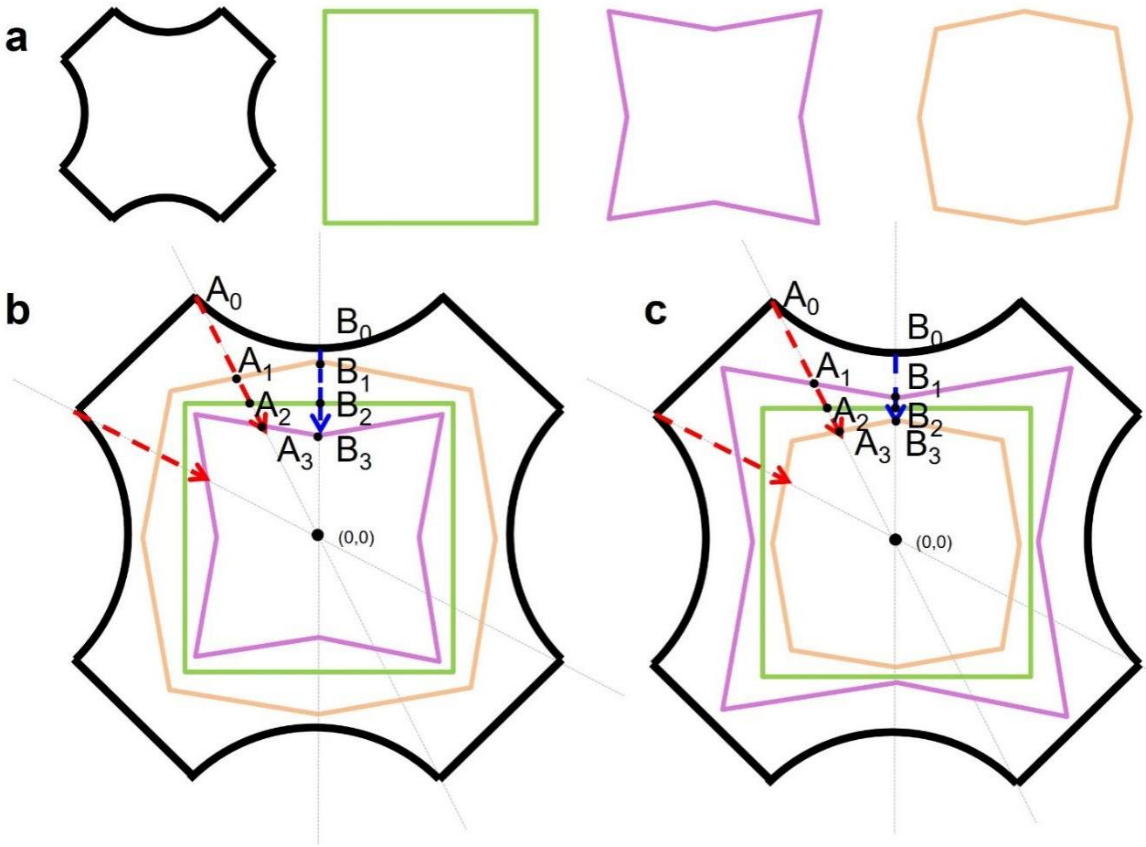

Supplementary Fig. 4 | Schematic illustration of pore shape evolution for imprinted nanoconcaves during anodization. (**a**) The black polygon represents a nanoconcave on the aluminium foil surface. The green square, the pink square with internally-bent walls, and the orange square with externally-bent walls are possible shapes of anodized pores, featuring fourfold symmetry. (**b**,**c**) Two possible trends of pore shape evolution as a function of AV. In (**b**), increasing AV leads to the pore shape alteration from the square with externally-bent walls to the square without bending walls and then the square with internally-bent walls; in (**c**), the pore shape is altered from the square with internally-bent walls to the square without bending walls and then the square with externally-bent walls. The red arrow marks the expansion pathways for two representative points (A_0 and B_0) as increasing AV. Two points have the maximum and the minimum electric fields along the nanoconcave wall, respectively (Supplementary Fig. 3).

Given that the nanoconcave shape and the corresponding electric field maps (Supplementary Fig. 3) are characterized by fourfold symmetry, the anodized pores will thus inherit the identical structural symmetry as the nanoconcave. The possible pore shapes featuring fourfold symmetry can be roughly categorized into three types: the square without bending walls, the square with internally-bent walls, and the square with externally-bent walls, as summarized in Supplementary Fig. 4a.

Previous reports pointed out that acid anions are driven into the oxide layer with the assistance of high electric fields, and the volume expansion is positively determined by the number of

incorporated acid anions[1,2]. Given that the A_0 point is always higher than the B_0 position in electric field (Supplementary Fig. 3), more acid anions will be incorporated into the A_0 point, leading to larger volume expansion than the B point. In particular, the AV increase from 120 V to 200 V also leads to greater electric field difference between the A_0 point and the B_0 point (Supplementary Fig. 3), which consequently causes larger volume expansion difference. As a result, the pore shape evolution as a function of AV shown in Supplementary Fig. 4b is not correct because its volume expansion difference gets reduced with the AV increase, namely,

$$\frac{A_0 \to A_1}{B_0 \to B_1} > \frac{A_0 \to A_2}{B_0 \to B_2} > \frac{A_0 \to A_3}{B_0 \to B_3}$$

where $X_i \rightarrow X_j$ is the distance between the point X_i and the point X_j.

Therefore, with the AV increase the pore shape should be altered from square with internally-bent walls to square without bending walls and then square with externally-bent walls, as schematically illustrated in Supplementary Fig. 4c. In such an alteration, the volume expansion difference between the points A and B is gradually enhanced, that is,

$$\frac{A_0 \to A_1}{B_0 \to B_1} < \frac{A_0 \to A_2}{B_0 \to B_2} < \frac{A_0 \to A_3}{B_0 \to B_3}$$

Accordingly, it is envisaged that higher AVs lead to smoother and more externally-bent walls and lower AVs lead to sharper and more internally-bent walls.

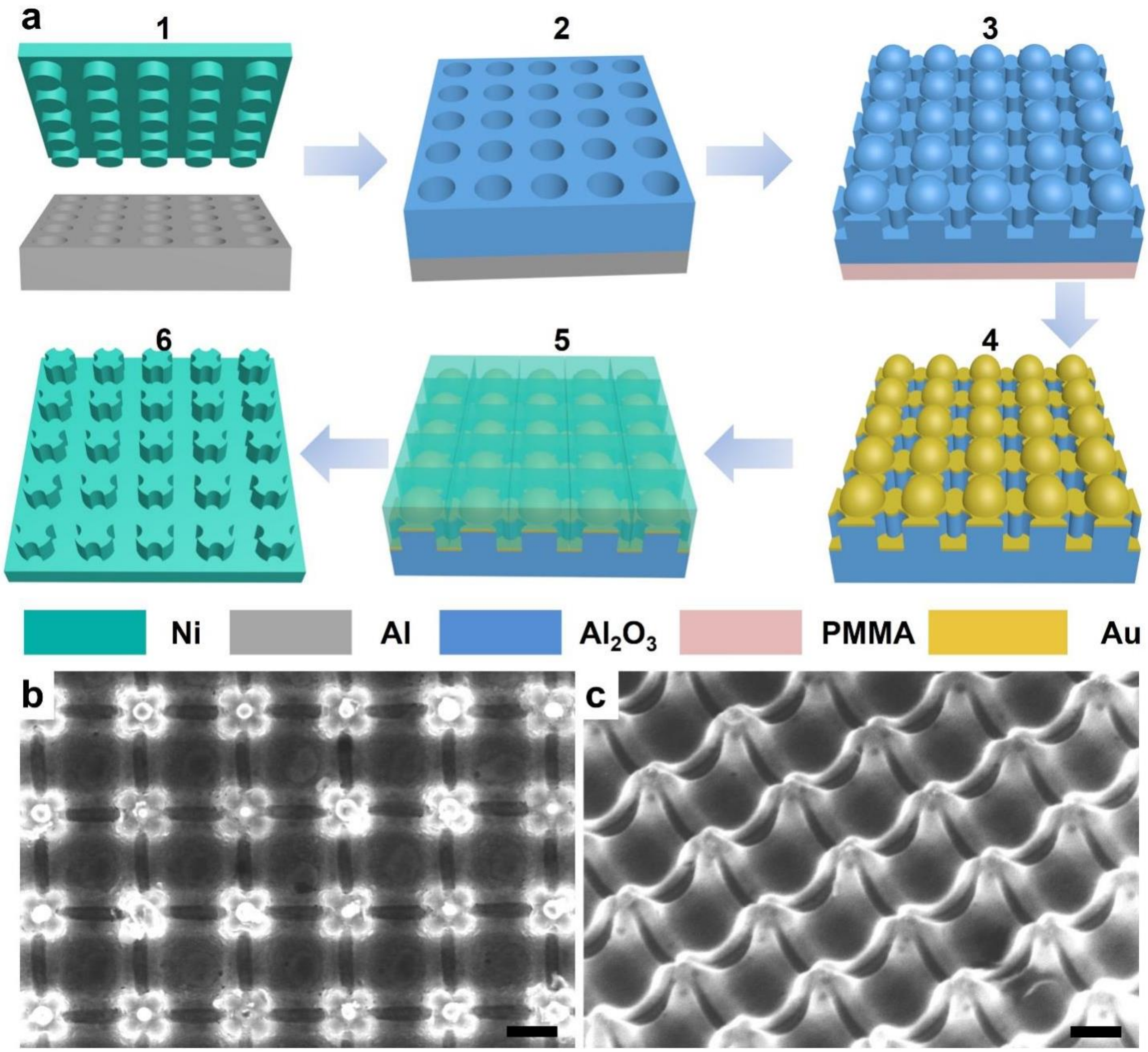

Supplementary Fig. 5 | Fabrication of Ni imprint stamp decorated with four-leaf clover-like nanopillars. (**a**) Schematic illustration of the fabrication process: (1) Imprinting aluminium foil using Ni stamp with a tetragonal array of circular nanopillars; (2) Anodic anodization; (3) Coating PMMA over the anodized area, followed by removing the unanodized aluminium and selectively etching the bottom surface of the anodized template; (4) Evaporating Au by PVD to form a conductive layer; (5) Performing Ni electrodeposition to replicate the as-etched shape of the bottom surface; (6) Dissolving PMMA and removing AAO template. (**b**) Top- and (**c**) tilted-view SEM images of Ni imprint stamp with four-leaf clover-like nanopillars. Because the tetragonal array of Ni nanopillars exploited in step 1 is 400 nm in spacing, the resultant array of four-leaf clover-like nanopillars obtained in step 6 is 400 nm as well. Scale bars: 200 nm.

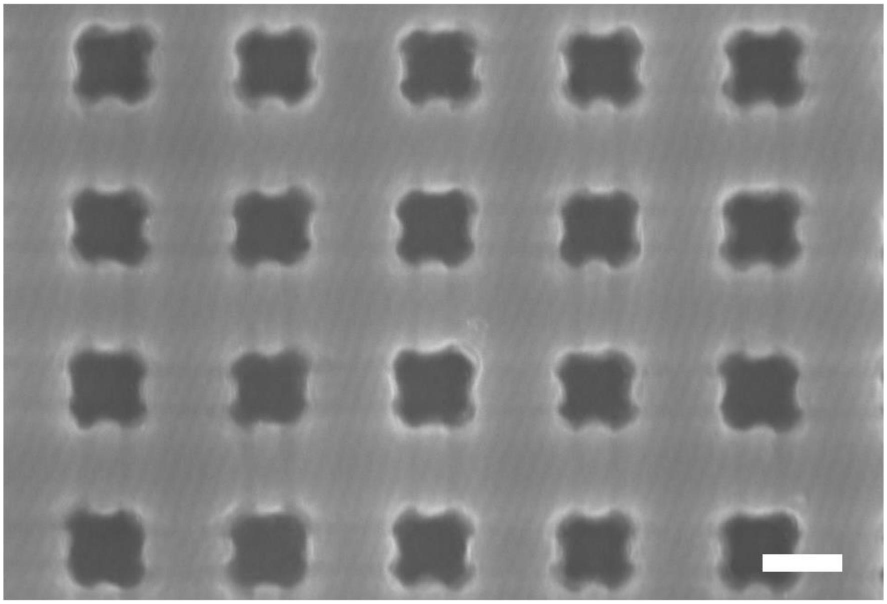

Supplementary Fig. 6 | Aluminium foil with a tetragonal array of four-leaf clover-like nanoconcaves. Scale bar: 200 nm.

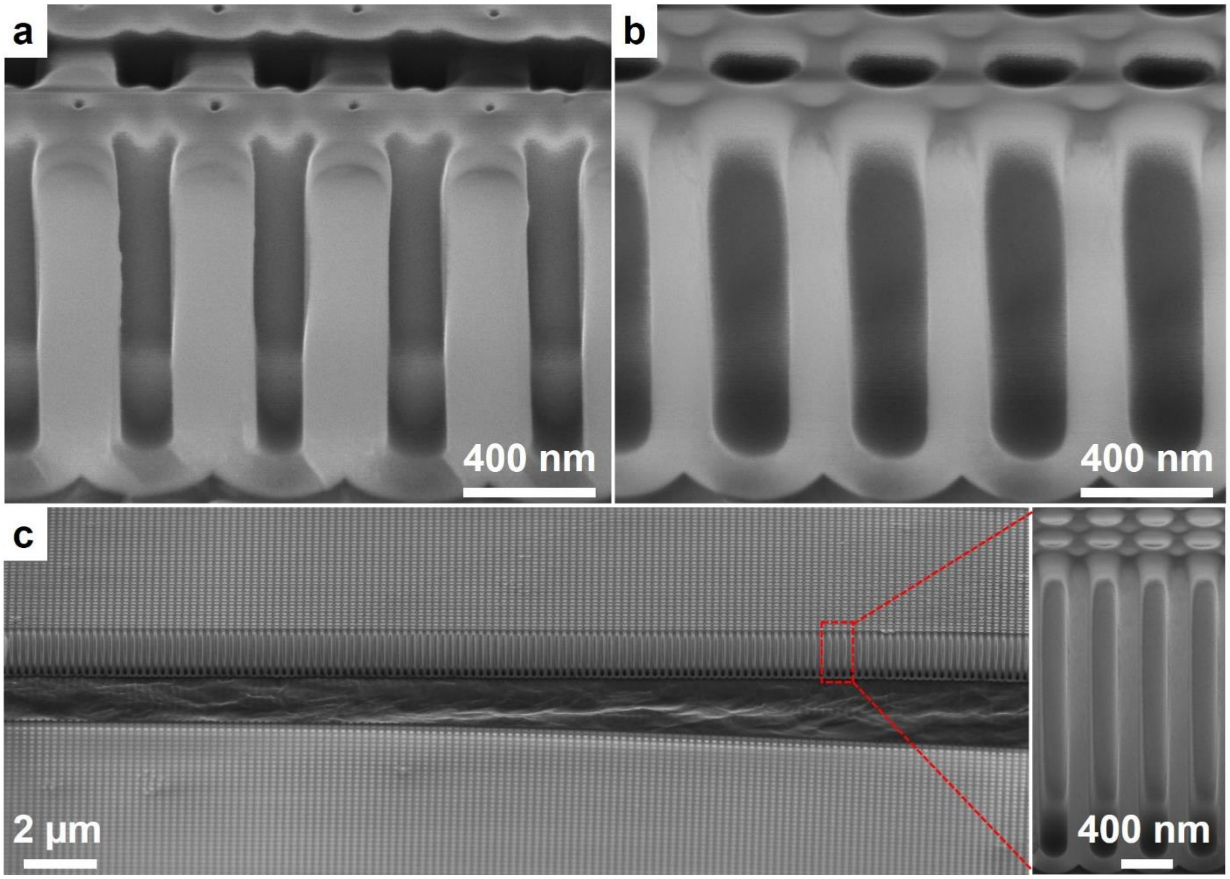

Supplementary Fig. 7 | Cross-sectional SEM images of tetragonally arranged nanopores anodized at different AVs. Anodization was carried out at (**a**) 120 V and (**b,c**) 200 V. For clearly showing the circular shape, pore-widening in NaOH solutions was performed upon the samples in (**b**,**c**).

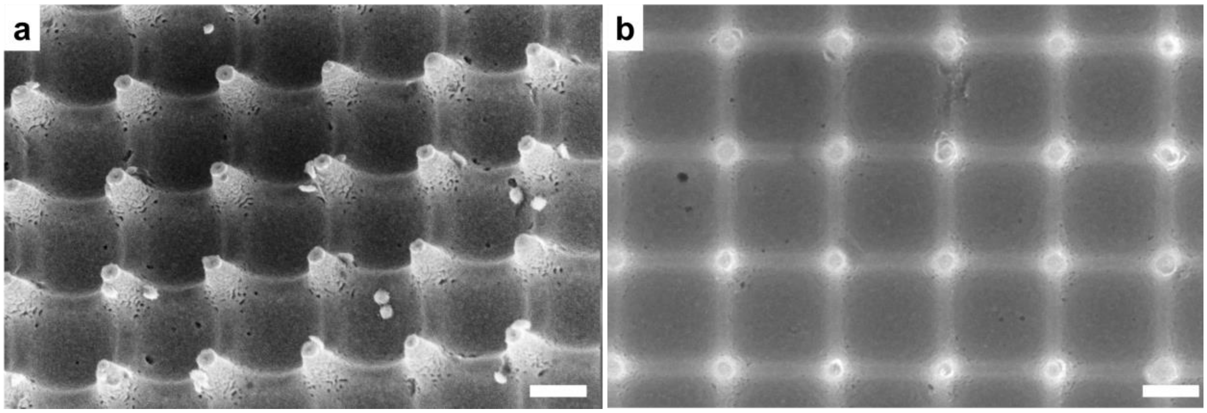

Supplementary Fig. 8 | Ni imprint stamp with circular nanopillars. (**a**) Tilted-view and (**b**) top-view SEM images of Ni circular nanopillars. Scale bars: 200 nm.

The fabrication process for the Ni stamp with circular nanopillars was almost the same as that of the Ni stamp with four-leaf clover-like nanopillars in Supplementary Fig. 5, except for the etching time of the AAO template in H_3PO_4 solutions (Step 3). To obtain circular nanopillars, the etching time was about 10 min.

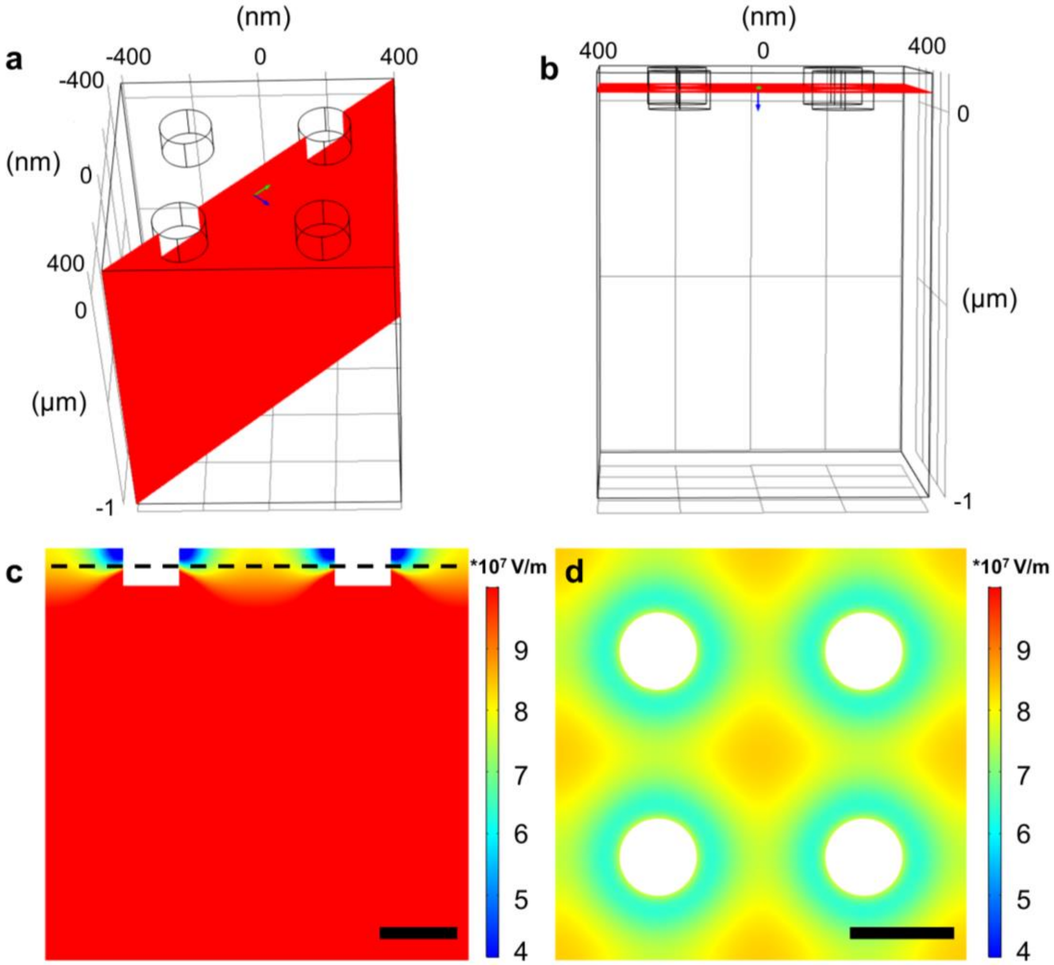

Supplementary Fig. 9 | COMSOL simulation of electric field distribution in Al foil patterned with a tetragonal array of circular nanodents. 3D layouts of surface-patterned Al foil with (**a**) vertical cross section and (**b**) near-surface lateral cross section. Simulated electric field maps across the (**c**) vertical cross section and (**d**) near-surface lateral cross section under an AV of 120 V. The dashed line in (c) at the half-depth of nanodents marks the near-surface lateral cross section in (d). Scale bars: 200 nm.

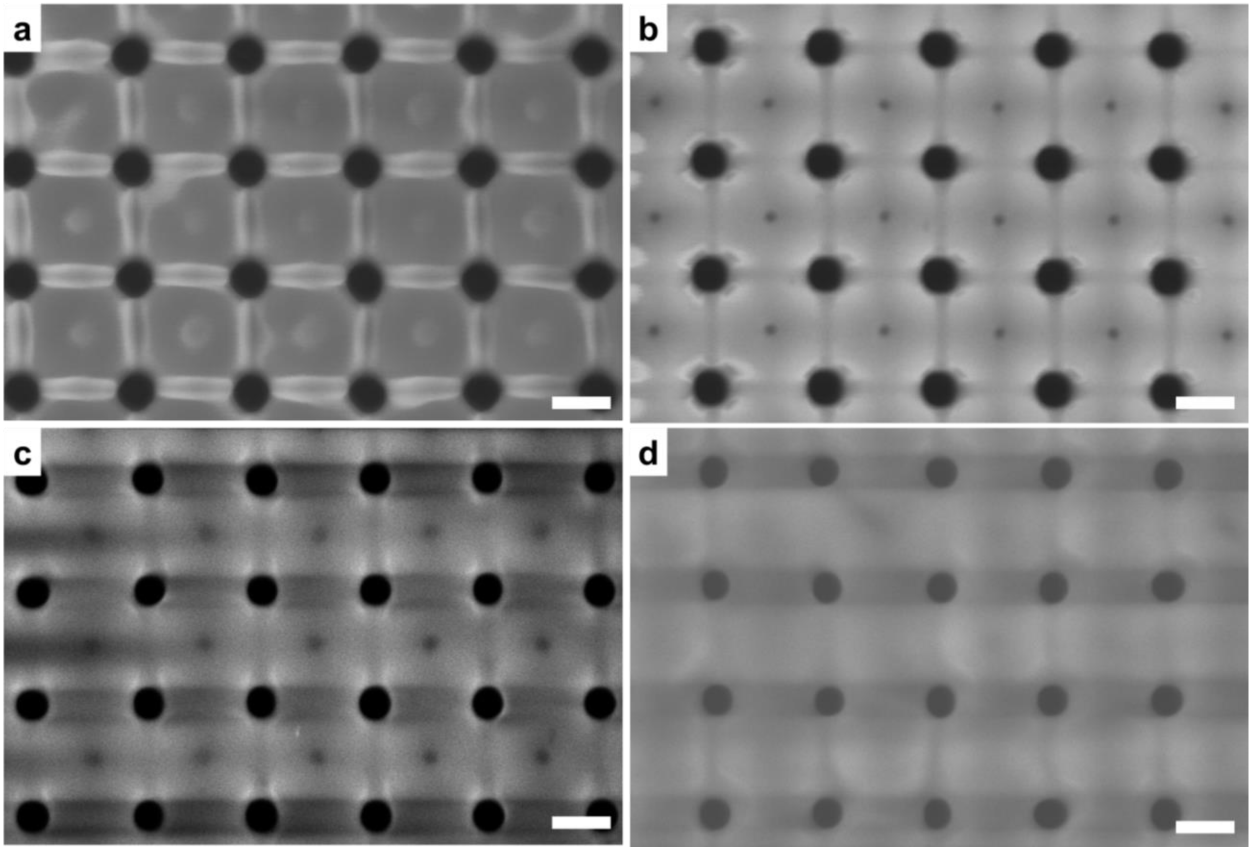

Supplementary Fig. 10 | AAO templates anodized at different AVs. Top-view SEM images of AAO templates (imprinted by the Ni stamp in Supplementary Fig. 8) anodized at (**a**) 120 V, (**b**) 140 V, (**c**) 160 V, and (**d**) 200 V, respectively. Scale bars: 200 nm.

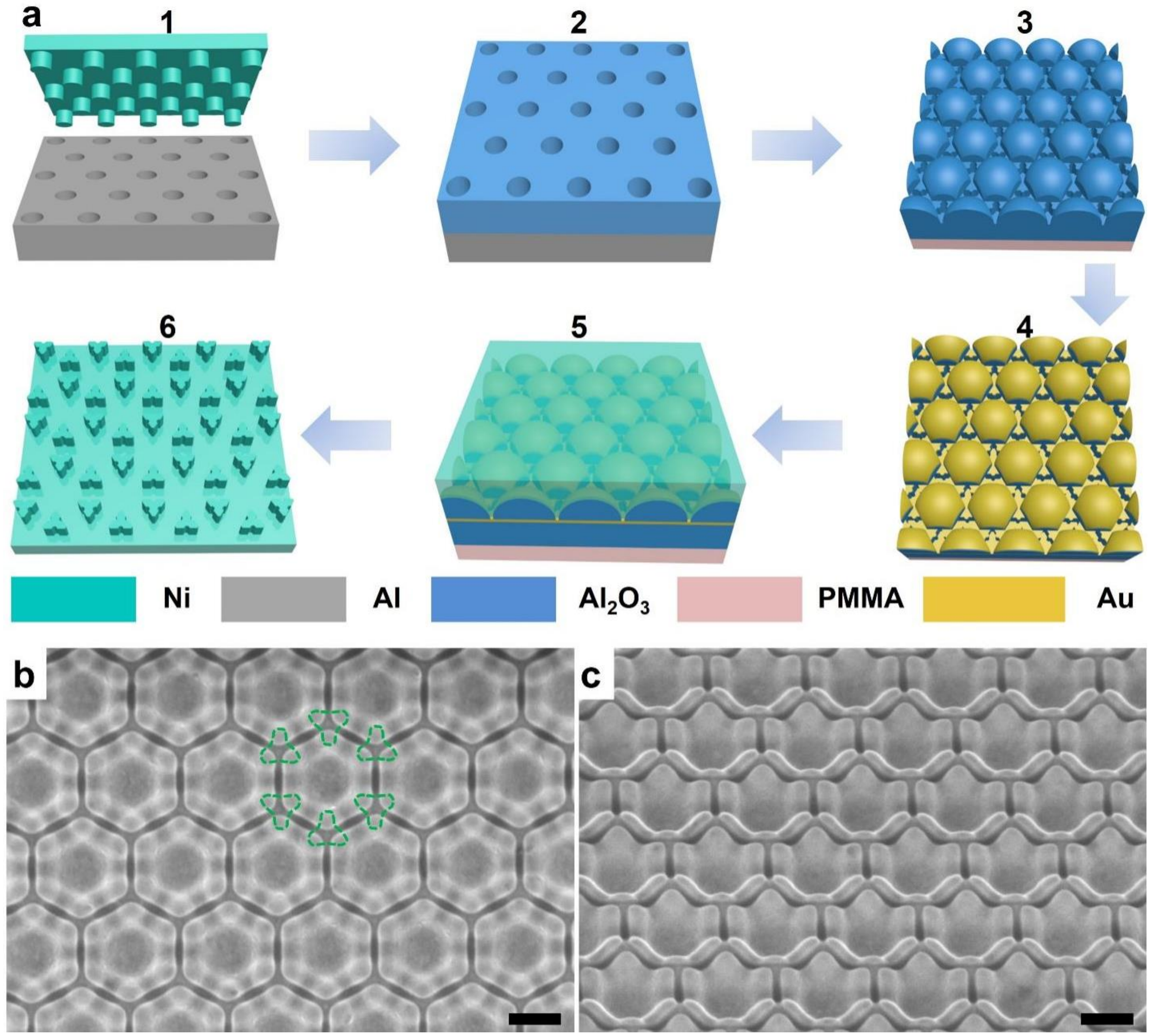

Supplementary Fig. 11 | Fabrication of Ni imprint stamp decorated with three-leaf clover-like nanopillars of hexagonal arrangement. (**a**) Schematic illustration for fabrication process. (**b**) Top-view and (**c**) tilted-view SEM images of Ni imprint stamp with three-leaf clover-like nanopillars. The shapes of nanopillars are outlined by green dashed lines in (**b**). Because the trigonal array of nanopillars anchored on the original Ni imprint stamp exploited in step 1 is 400 nm in spacing, the resultant hexagonal array of three-leaf clover-like nanopillars obtained in step 6 is $400/\sqrt{3}$ nm. Scale bars: 200 nm.

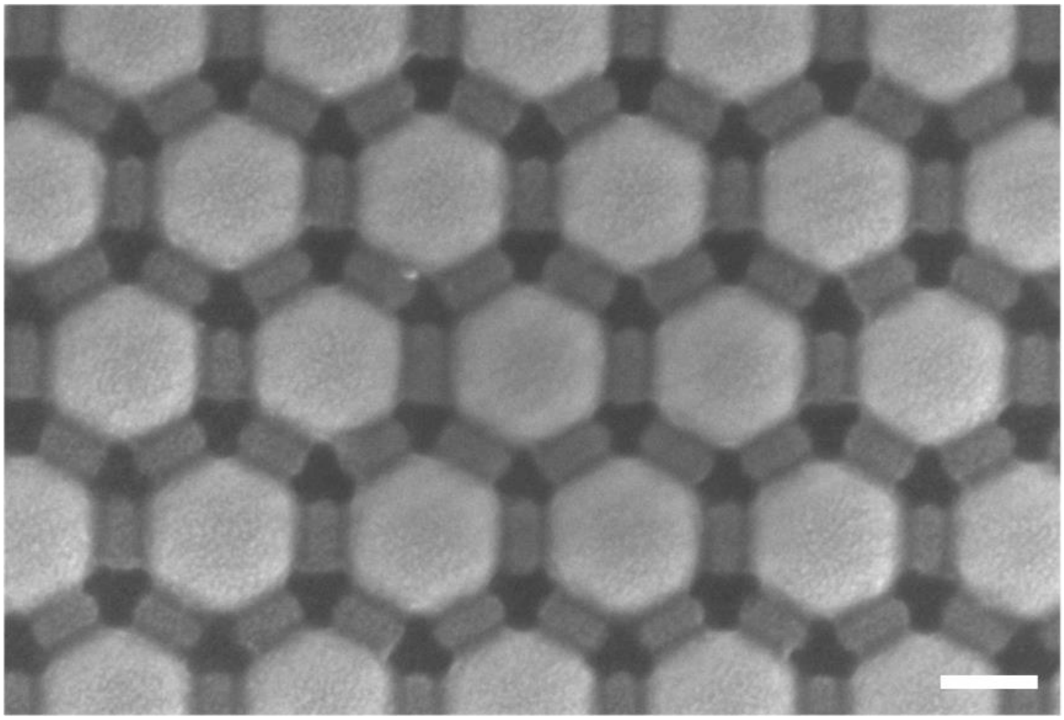

Supplementary Fig. 12 | Surface-patterned aluminium foil with a hexagonal array of three-leaf clover-like nanoconcaves. Scale bar: 200 nm.

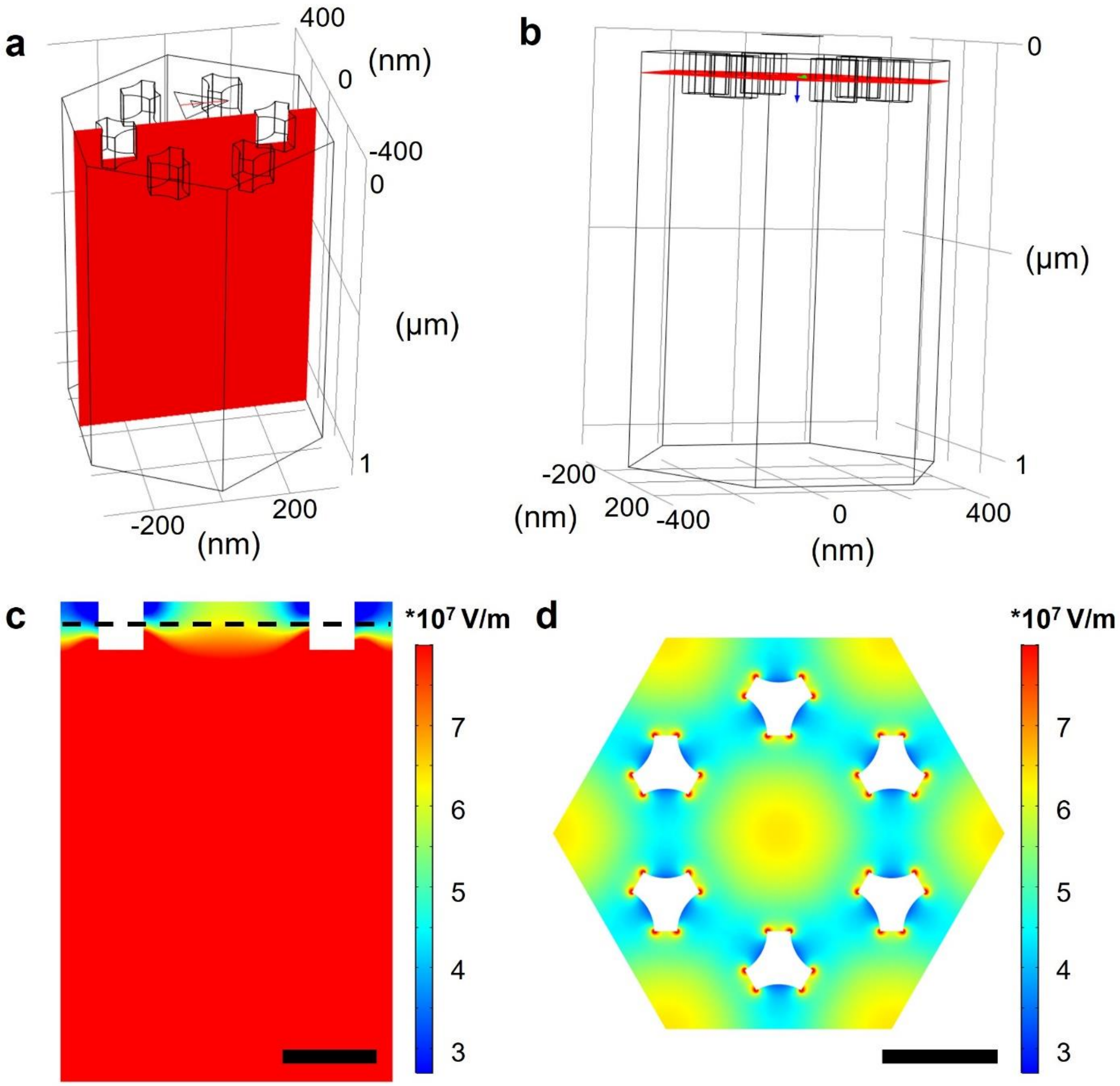

Supplementary Fig. 13 | Simulation of electric field maps in an aluminium foil engineered with a hexagonal array of three-leaf clover-like nanoconcaves at the initial stage of anodization. (**a**) Vertical cross section and (**b**) near-surface lateral cross section of three-dimensional surface-patterned aluminium foil. Simulated electric field maps under an AV of 100 V at the (**c**) vertical cross section and (**d**) near-surface lateral cross section. The dashed line in (**c**) at the half-depth of nanoconcaves indicates the near-surface lateral cross section in (**d**). Scale bars: 200 nm.

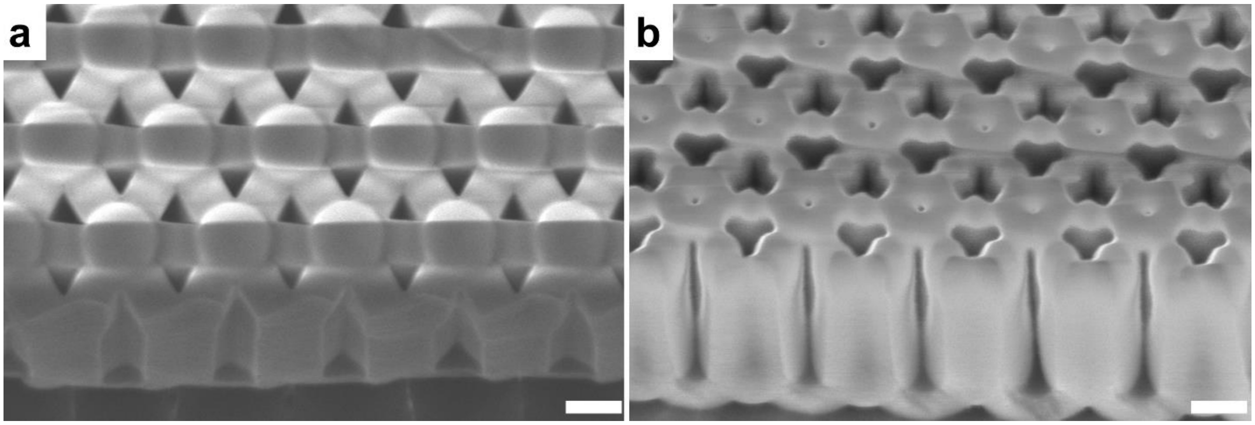

Supplementary Fig. 14 | Cross-sectional SEM images of hexagonally arranged nanopores anodized at different AVs. Anodization was carried out at (**a**) 140 V and (**b**) 100 V, respectively. Scale bars: 200 nm.

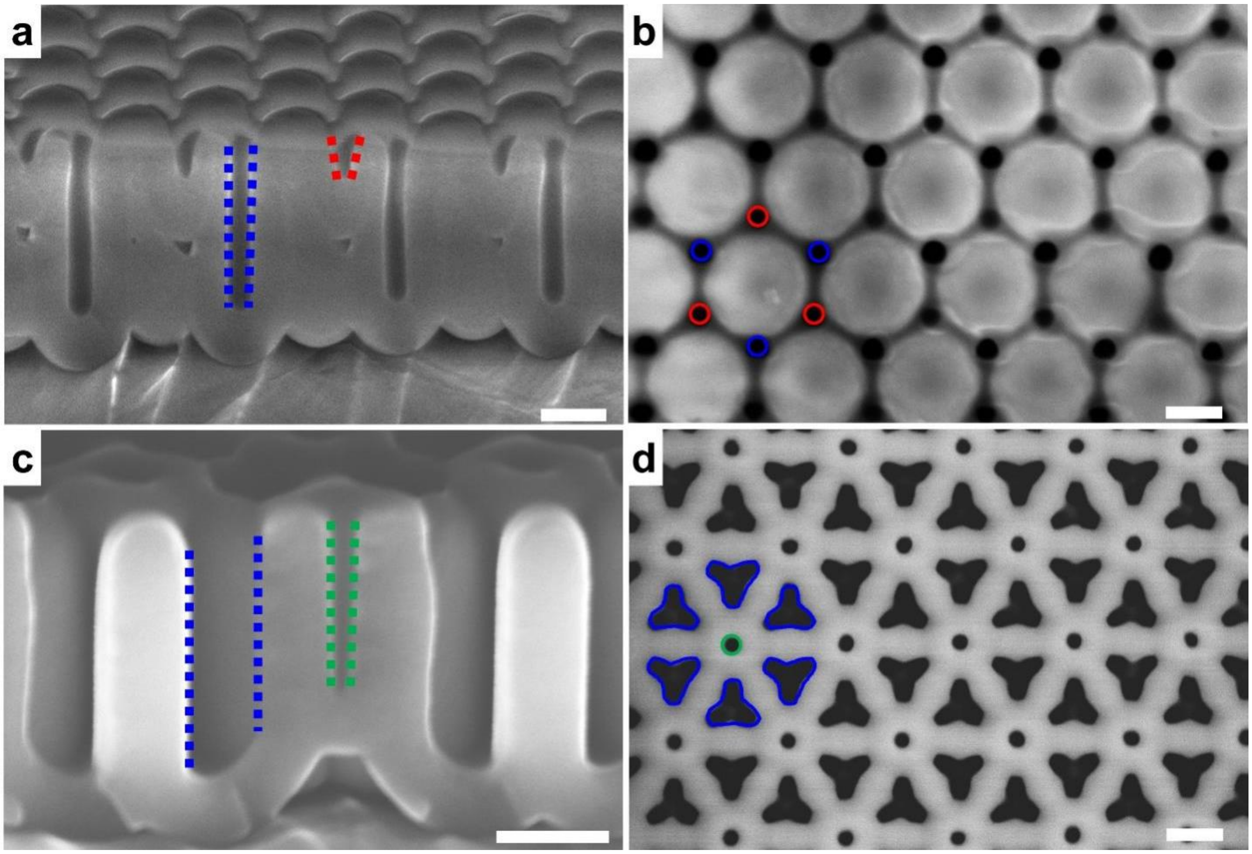

Supplementary Fig. 15 | AAO templates obtained by anodizing aluminium foil with a hexagonal array of three-leaf clover-like nanoconcaves in a broad AV range. (**a**) Cross-sectional-view and (**b**) top-view SEM images of an AAO template anodized at 180 V, showing that within a hexagonal unit of six pores, three diagonally neighboring pores (red-colored) disappear, leaving only very shallow nanoconcaves on the top of the template. (**c**) Cross-sectional-view and (**d**) top-view SEM images of an AAO template anodized at 80 V, showing that a new pore (green-colored) was formed at the junction site of six adjacent pores. Scale bars: 200 nm.

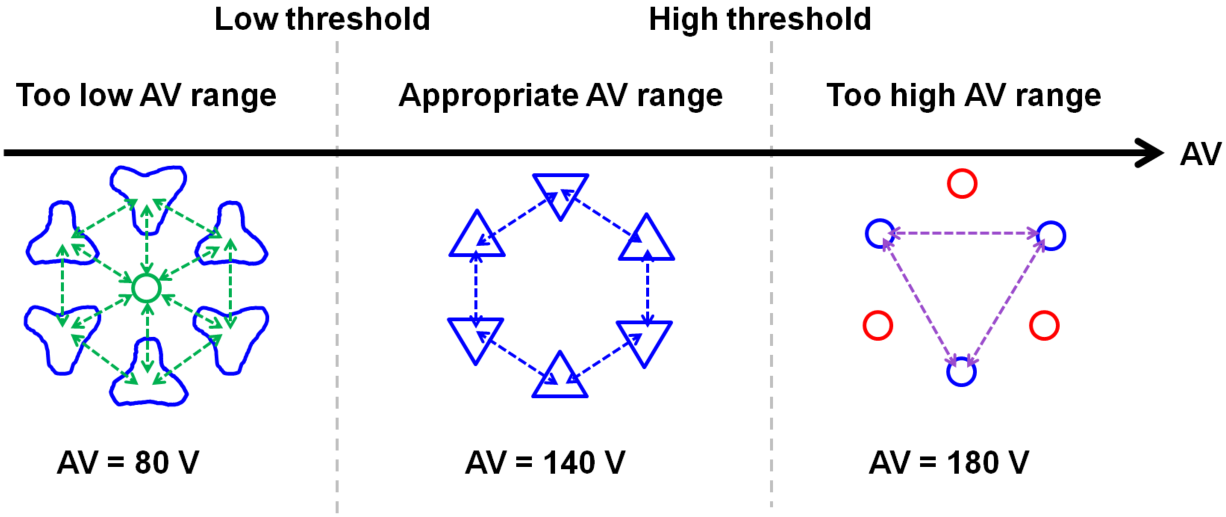

Supplementary Fig. 16 | Pore arrangement after anodization at different AVs. Here we select the hexagonal array of nanoconcaves as an example and demonstrate three representative pore arrangements after anodization with the AVs of 80, 140, and 180 V.

Based on the experimental results shown in Fig. 2e-h and Supplementary Fig. 15, it can be concluded that the AVs for a specific array of nanoconcaves should be divided into three ranges: too low AV range, appropriate AV range, and too high AV range. For appropriate AVs, the pore arrangement (*e.g.*, the hexagonal arrangement depicted by blue dashed arrows) predetermined by imprinting can remain during anodization; for too high AVs, the predetermined arrangement was destroyed due to the disappearance of diagonally neighboring red pores, forming a new trigonal arrangement (depicted by purple dashed arrows); for too low AVs, the pre-set arrangement was also destroyed with the occurrence of a green pore at the center of a unit cell (*i.e.*, the junction site of six adjacent blue-colored pores), giving rise to another trigonal arrangement (depicted by green dashed arrows).

In general, the three ranges are separated by two threshold values, as schematically illustrated in Supplementary Fig. 16. Its two thresholds are empirically observed to be V_a and $\sqrt{3}V_a$, where V_a = 0.4 nm/V × L_h and L_h is the interpore spacing of the hexagonal array (*i.e.*, 400/$\sqrt{3}$nm). In other words, AV thresholds can be derived from the linear spacing-AV relation, *i.e.*, AV (V) = spacing (nm) × 0.4 (V/nm) or spacing (nm) = AV (V) ×2.5 (nm/V) [3], regarding two spacings (*e.g.*, L_h and $\sqrt{3}L_h$) of emerging arrays.

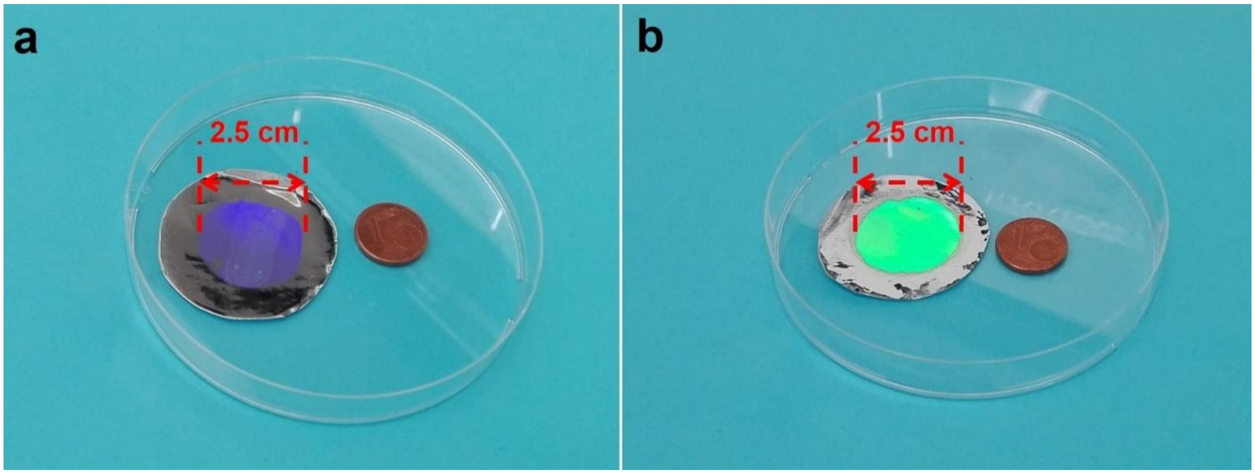

Supplementary Fig. 17 | Optical photographs of surface-patterned aluminium foils (a) before and (b) after anodization.

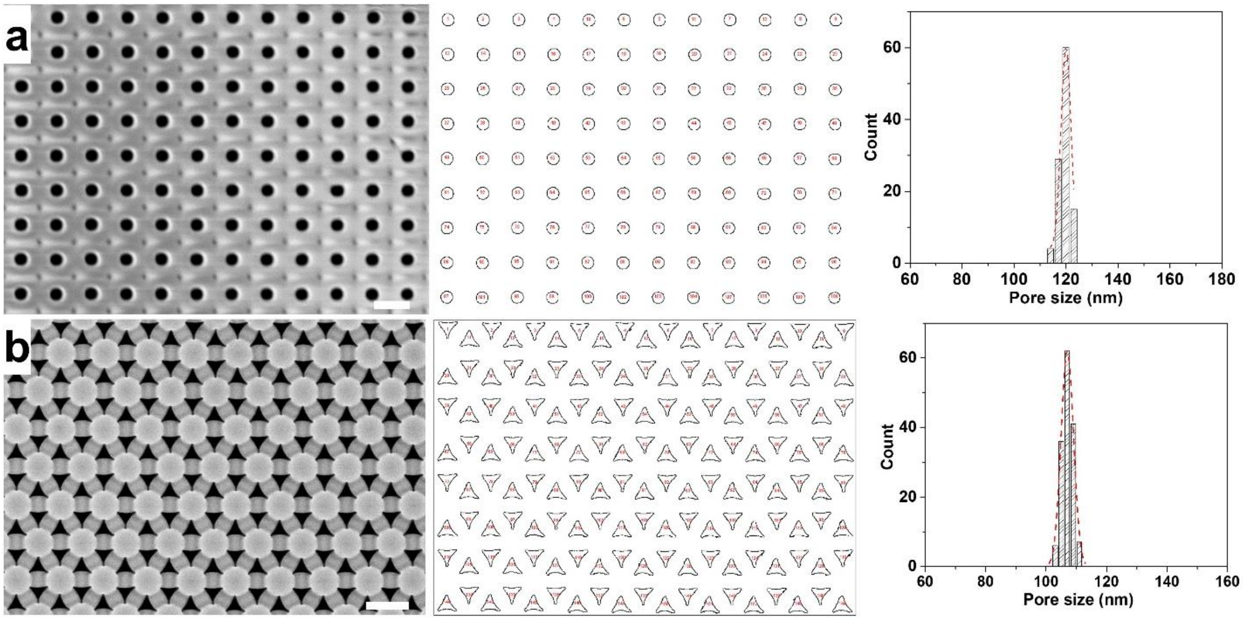

Supplementary Fig. 18 | Nearly monodisperse pore size distribution of AAO template. Pores are arrayed in (**a**) tetragonal and (**b**) hexagonal arrangements. Scale bars: 400 nm.

Given the shape diversity for pores in AAO templates, especially for those with bent walls, there is a lack of a universal parameter to evaluate pore size. For quantitative comparison, the pore size (*Size*) in this work was calculated by

$$Size = \sqrt{area} \tag{1}$$

Statistical analysis of pore area (*area*) was performed with open-source software ImageJ (downloaded from https://imagej.nih.gov/ij/index.html). The slight deviation was mostly ascribed to the geometrical dispersion of the lab-made Ni nanopillars and should be avoided using standard imprint stamps.

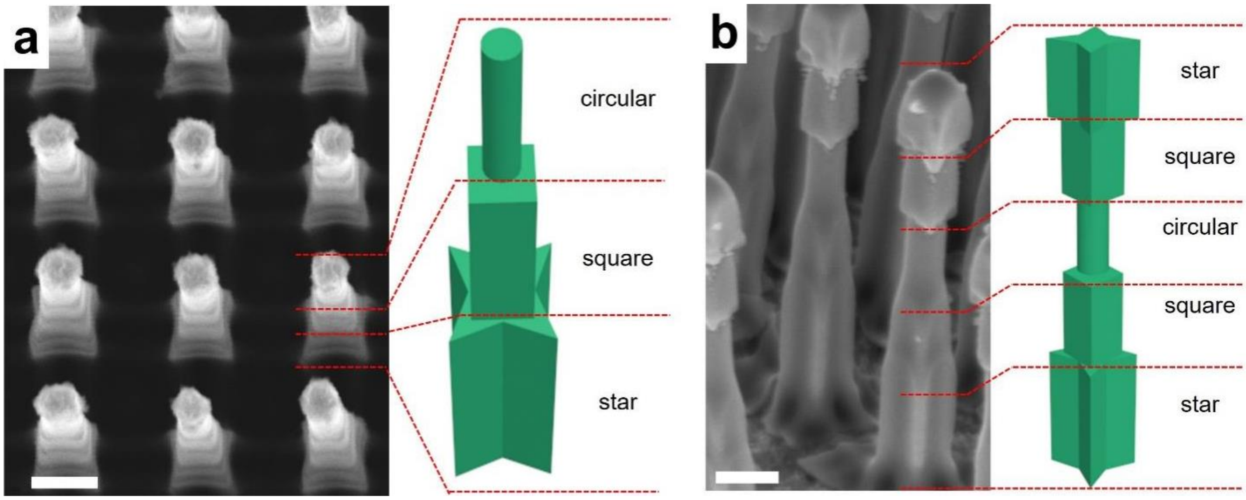

Supplementary Fig. 19 | TiO_2 multi-segment nanowires. (**a**) Tilted-view SEM image and schematic illustration for three-segment nanowires (from bottom to top: star+square+circular), replicated from an AAO template with three-segment pores (sequentially anodized at 140→160→200 V). (**b)** Tilted-view SEM image and schematic illustration for five-segment nanowires (from bottom to top: star+square+circular+square+star), replicated from an AAO template with five-segment pores (sequentially anodized at 140→160→200→160→140 V). Scale bars: 200 nm.

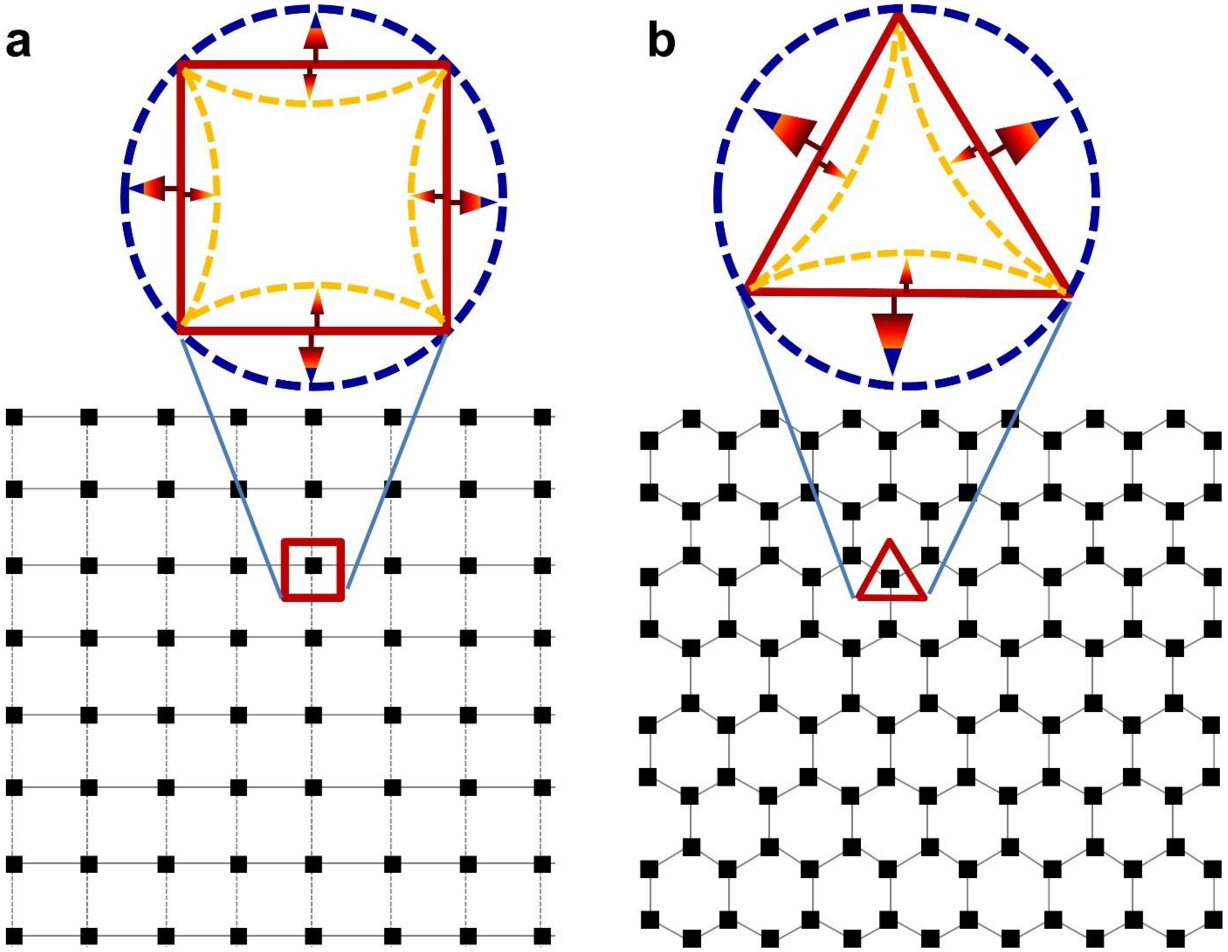

Supplementary Fig. 20 | Shape tuning trend (up) of AAO pores in a specific spatial configuration (down). The black dots represent the central sites of pores in the corresponding arrangements. The blue, red, and yellow polygons schematically outline the pore shapes anodized at different AVs.

(**a**) In the tetragonal arrangement, the pore can be evolved from the square shape to the wall-externally-bent square shape by increasing AV or to the wall-internally-bent square shape by decreasing AV. (**b**) In the hexagonal arrangement, the pore shape can be evolved from the triangular shape to the wall-externally-bent triangular shape by increasing AV or to the wall-internally-bent triangular shape by decreasing AV.

In general, the walls of the as-anodized pores are always perpendicular to the lines that link the central sites of two adjacent pores. That is to say, the pore shape in term of structural symmetry is highly dependent on the spatial configuration of neighboring pores.

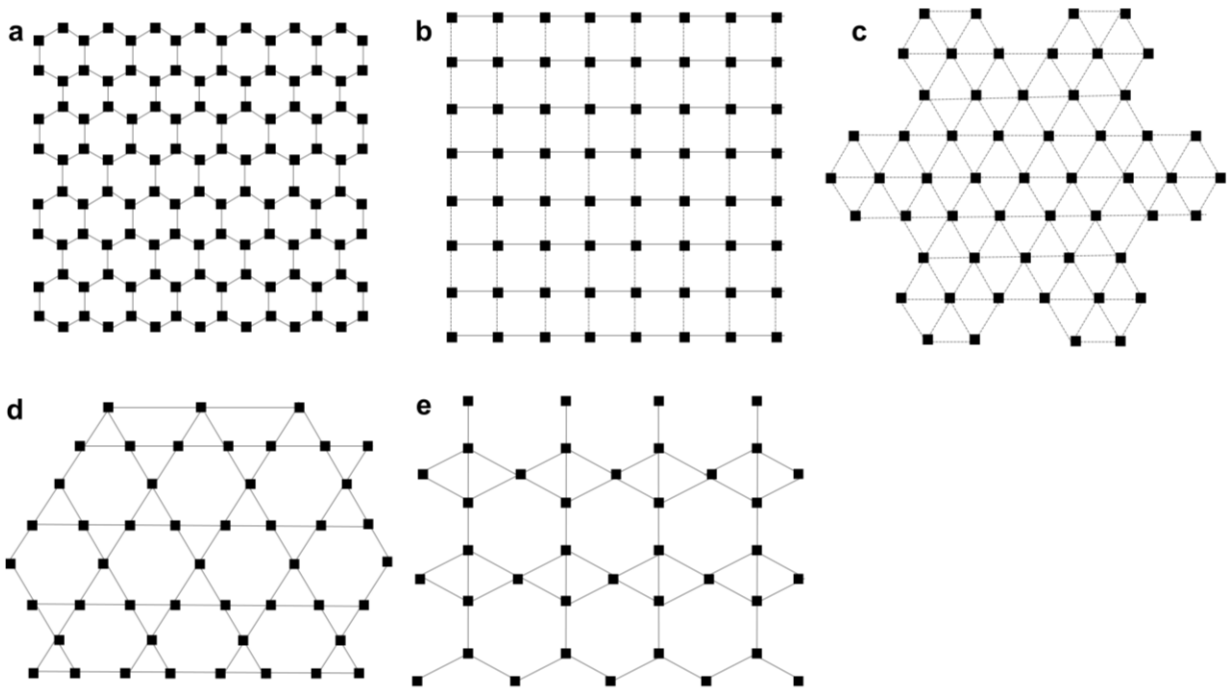

Supplementary Fig. 21 | Tessellation of a two-dimensional plane using regular polygons: (**a**) hexagon, (**b**) tetragon, (**c**) trigon, and (**d**,**e**) combination of hexagon and trigon. The black dots represent the central sites of pores in the corresponding arrangements.

If a tessellation is tiled without gap by only one regular polygon, the corners of the polygon need to fit into each other around a point, meaning that 360° must be evenly divided by the corner angle (β) of the regular polygon:

$$\frac{360^o}{\beta} = n \qquad (2)$$

where n are integers (*e.g.*, 1, 2, 3…).

For the regular polygon including m sides, the corner angle β can be described by the following equation:

$$\beta = \frac{(m-2)\times 180^o}{m} = 180^o - \frac{360^o}{m} \qquad (3)$$

Because of $m \geq 3$, the corner angle β is never less than 60°.

Based on the above recognition, we can conclude that:

(i) when n = 3, β = 120°, 3 × 120° = 360°, there is a tessellation using three regular hexagons around each vertex, corresponding to the hexagonal arrangement shown in Supplementary Fig. 21a;

(ii) when n = 4, β = 90°, 4 × 90° = 360°, there is a tessellation using four regular tetragons around each vertex, corresponding to the tetragonal arrangement shown in Supplementary Fig. 21b;

(iii) when n = 6, β = 60°, 6 × 60° = 360°, there is a tessellation using six regular trigons around each vertex, corresponding to the trigonal arrangement shown in Supplementary Fig. 21c.

(iv) if n is further increased (*e.g.*, n = 9, 12, 18), the corner angle β (*e.g.*, 40°, 30°, 20°) will be less than 60°.

Therefore, only three regular polygons (*i.e.*, trigon, tetragon, and hexagon) can tile a two-dimensional plane without gap.

Of course, different regular polygons with the same side-length also can be combined for tessellation of a two-dimensional plane, such as the combination of hexagon and trigon shown in Supplementary Fig. 21d, e which accord with the spatial configuration of pores in Fig. 3a,d of Ref. 4. In order to follow the linear conventional linear spacing-AV relation[3], it was believed that the interpore spacings for different arrangements in the AAO template should be identical.

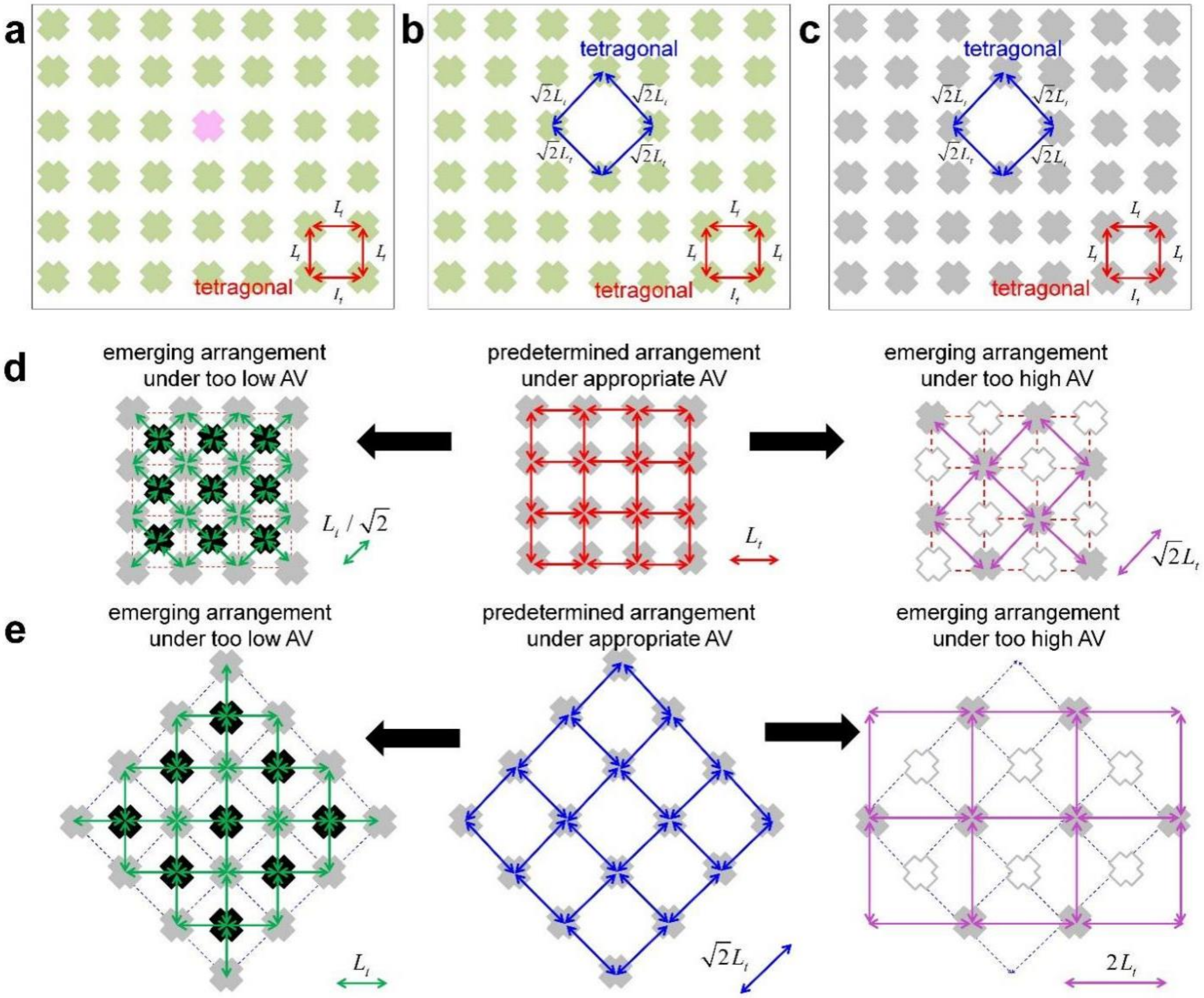

f

Arrangement	Low AV threshold	High AV threshold
Basic tetragonal arrangement	$V_b / \sqrt{2}$	$\sqrt{2}V_b$
Accessory larger-spacing tetragonal arrangement	V_b	$2V_b$
Mixture arrangement	V_b	$\sqrt{2}V_b$

Supplementary Fig. 22 | Pore arrangement evolution of surface-patterned aluminium foil with spacing-different tetragonal arrangements under different AVs. Schematic illustrations for (**a**) imprint stamp of L_t-spacing tetragonal arrangement and (**b**) imprint stamp equipped with two tetragonal arrangements of different spacings (*i.e.*, L_t and $\sqrt{2}L_t$), L_t = 400 nm. For obtaining the imprint stamp in (**b**), a nanopillar marked by pink color in (A) was removed by using Zeiss FIB-SEMs system. (**c**) Surface-patterned aluminium foil using imprint stamp in (B). Pore arrangement evolution of (**d**) basic L_t-spacing tetragonal arrangement and (**e**) accessory $\sqrt{2}L_t$-spacing tetragonal arrangement under too low AVs, appropriate AVs, and too high AVs. The pores shown in (**d**) and (**e**) are just for demonstration of pore arrangement and do not represent

the real pore shapes. The gray cross, black cross, and hollow cross demonstrate the pores occurring at the site of predetermined nanoconcave, the emerging pores, and the disappearing pores from the sites of predetermined nanoconcaves, respectively. (**f**) Appropriate AV ranges for the basic L_t-spacing tetragonal arrangement, the accessory $\sqrt{2}L_t$-spacing tetragonal arrangement and the mixture arrangement.

As stated in Supplementary Fig. 16, the appropriate AV range for a specific arrangement should have two thresholds which are derived from the linear spacing-AV relation regarding two spacings of emerging arrays[3]. To prevent the disappearance of diagonally neighbouring pores during anodization for the L_t-spacing arrangement (middle part of Supplementary Fig. 22d), the appropriate AVs should be lower than $\sqrt{2}V_b$ (that is, the high AV threshold defined by 0.4 V/nm × $\sqrt{2}L_t$), where V_b corresponds to the conventional AV for the L_t-spacing arrangement and is defined by 0.4 V/nm × L_t. And $\sqrt{2}L_t$ indicates the spacing of emerging arrangement (depicted by pink arrows) after the disappearance of diagonally neighbouring pores from the L_t-spacing arrangement under too high AVs, as shown in the right part of Supplementary Fig. 22d. Similarly, the appropriate AVs for the $\sqrt{2}L_t$-spacing arrangement should be lower than $2V_b$ (that is, the high AV threshold defined by 0.4 V/nm × $2L_t$). And $2L_t$ indicates the spacing of emerging arrangement (depicted by pink arrows) after the disappearance of diagonally neighbouring pores from the $\sqrt{2}L_t$-spacing arrangement under too high AVs, as shown in the right part of Supplementary Fig. 22e.

Furthermore, to prevent the occurrence of new pores at the central site of a tetragonal unit during anodization for the L_t-spacing arrangement, the appropriate AVs for the L_t-spacing arrangement should be higher than $V_b/\sqrt{2}$, that is, the low AV threshold defined by 0.4 V/nm × $L_t/\sqrt{2}$,where $L_t/\sqrt{2}$ indicates the spacing of emerging arrangement (depicted by green arrows) after the occurrence of new pores in the L_t-spacing arrangement under too low AVs, as shown in the left part of Supplementary Fig. 22d. Similarly, the appropriate AVs for the $\sqrt{2}L_t$-spacing arrangement should be higher than V_b, that is, the high AV threshold defined by 0.4 V/nm × L_t. And L_t indicates the spacing of emerging arrangement (depicted by green arrows) after the occurrence of new pores in the $\sqrt{2}L_t$-spacing arrangement under too low AVs, as shown in the left part of Supplementary Fig. 22e.

In theory, the appropriate AV ranges for two arrangements should be ($V_b/\sqrt{2}$, $\sqrt{2}V_b$) and (V_b, $2V_b$), respectively. Thus, the intersectional AV range for the mixture arrangement should be from V_b (160 V) to $\sqrt{2}V_b$ (~226 V).

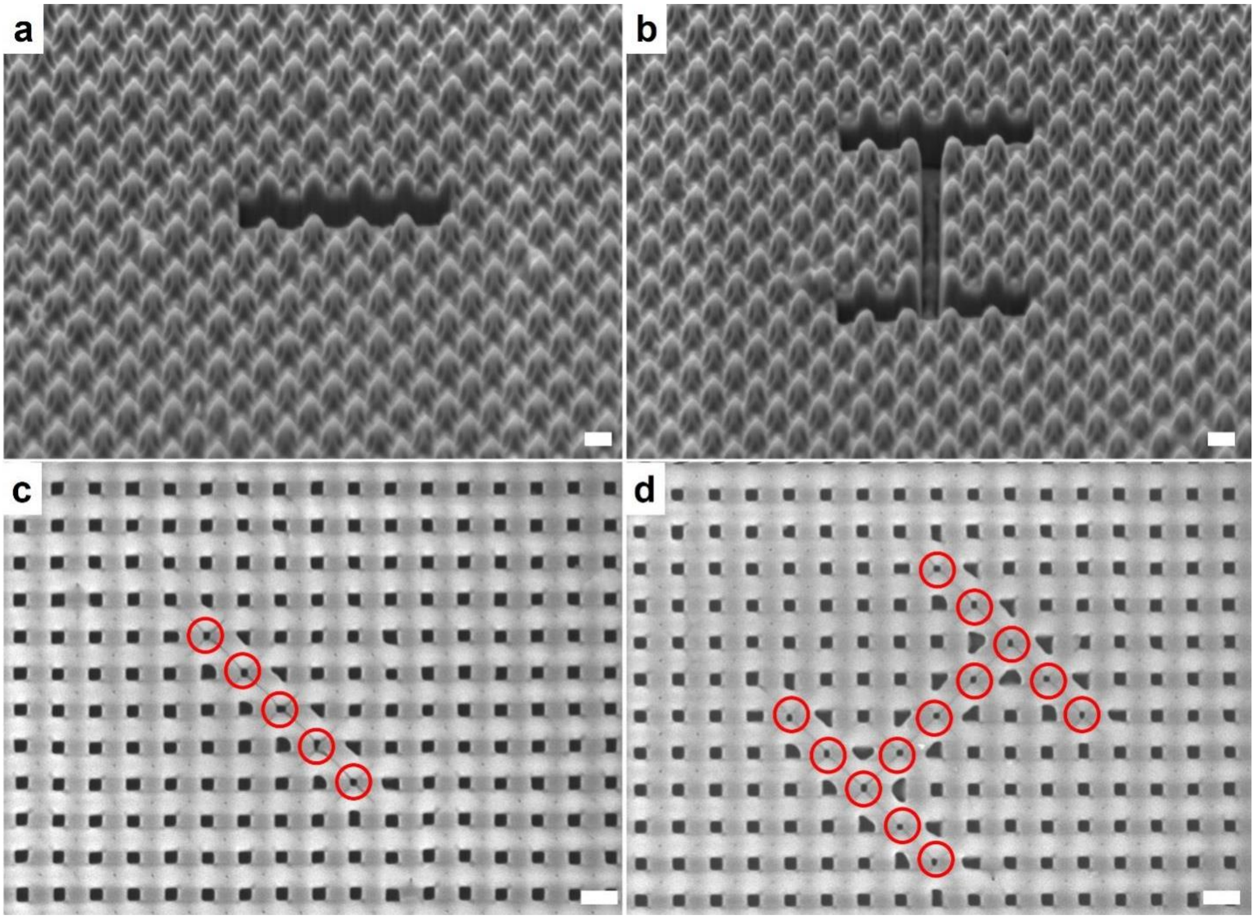

Supplementary Fig. 23 | Anodization upon surface-patterned aluminium foils with two spacing-different arrangements. (**a,b**) Several nanopillars in Ni perfect stamps were removed by Zeiss FIB-SEMs system to combine two arrangements in one matrix. Because the spacing of the basic arrangement is L_t = 400 nm, the new arrangement has the spacing of $\sqrt{2}L$. After performing anodization at 158 V, small pores were still obtained at the deficiency sites without nanoconcaves, as observed in (**c,d**) SEM images of AAO templates. Scale bars: 400 nm.

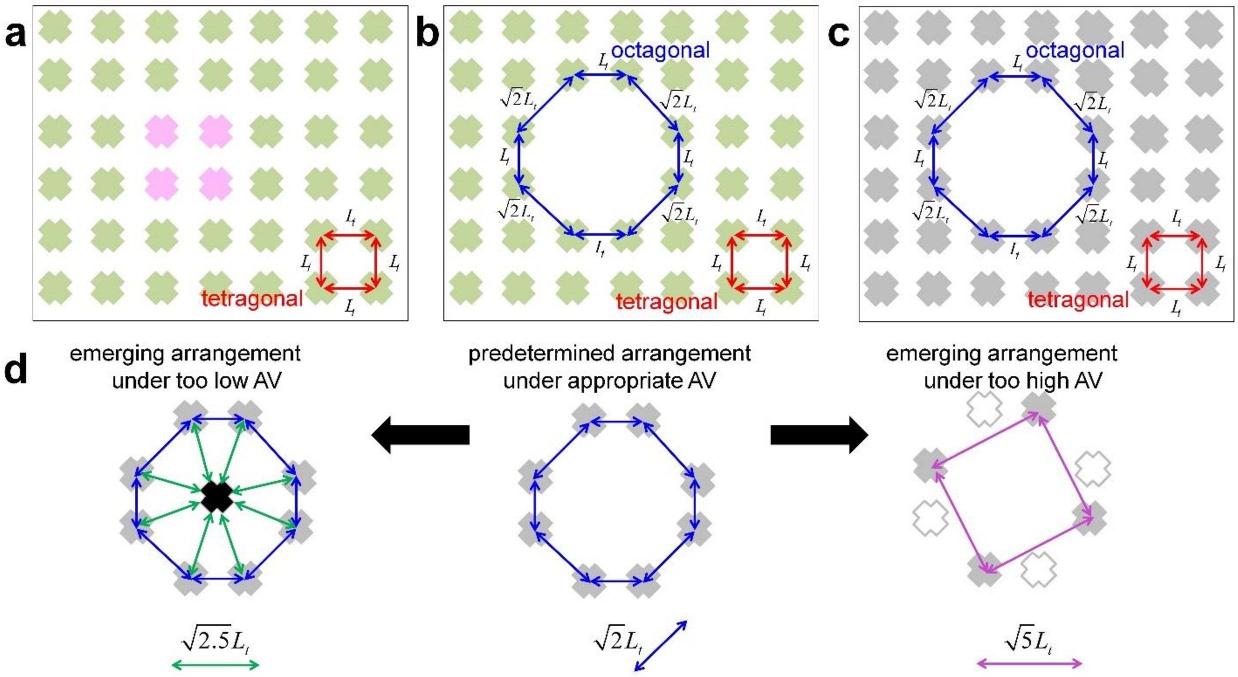

e

Arrangement	Low AV threshold	High AV threshold
Basic tetragonal arrangement	$V_b / \sqrt{2}$	$\sqrt{2}V_b$
Accessory larger-spacing tetragonal arrangement	$\sqrt{2.5}V_b$	$\sqrt{5}V_b$
Mixture arrangement	none	none

Supplementary Fig. 24 | Pore arrangement evolution of surface-patterned aluminium foils with mixture tetragonal and octagonal arrangements under different AVs. Schematic illustrations for (**a**) imprint stamp of tetragonal arrangement and (**b**) imprint stamp equipped with mixture tetragonal and octagonal arrangement. For obtaining the imprint stamp in (**b**), four neighboring nanopillars marked by pink color in (**a**) were removed using Zeiss FIB-SEMs system. (**c**) Surface-patterned aluminium foil using imprint stamp in (**b**). (**d**) Pore arrangement evolution of accessory octagonal arrangement under too low AVs, appropriate AVs, and too high AVs. The pores shown in (**d**) do not represent the real pore shapes. The gray cross, black cross, and hollow cross demonstrate the pore occurring at the site of predetermined nanoconcave, the emerging pore, and the disappearing pore from the site of predetermined nanoconcave, respectively. (**e**) Appropriate AV ranges for the basic L_t-spacing tetragonal arrangement, the accessory octagonal arrangement, and the mixture arrangement. L_t = 400 nm.

Following the same strategy introduced in Supplementary Fig. 22, to prevent the disappearance of diagonally neighboring pores during anodization for the octagonal arrangement (middle part of Supplementary Fig. 24d), the appropriate AVs should be lower than $\sqrt{5}V_b$ (that is, the high AV threshold defined by 0.4 V/nm × $\sqrt{5}L_t$), where V_b corresponds to the conventional AV for the

basic L_t-spacing tetragonal arrangement and is defined by 0.4 V/nm × L_t. And $\sqrt{5}L_t$ indicates the spacing of emerging arrangement (depicted by pink arrows) after the disappearance of diagonally neighboring pores from the octagonal arrangement under too high AVs, as shown in the right part of Supplementary Fig. 24d. Furthermore, to prevent the occurrence of a new pore at the central site of an octagonal unit during anodization, the appropriate AVs for the octagonal arrangement should be higher than $\sqrt{2.5}V_b$, that is, the low AV threshold defined by 0.4 V/nm × $\sqrt{2.5}L_t$ where $\sqrt{2.5}L_t$ indicates the distance between the emerging pore at the central site of an octagonal unit and an existing pore (depicted by green arrows) under too low AVs, as shown in the left part of Supplementary Fig. 24d.

In theory, the appropriate AV ranges for two arrangements should be ($V_b/\sqrt{2}$, $\sqrt{2}V_b$) and ($\sqrt{2.5}V_b$, $\sqrt{5}V_b$), respectively. Thus, there is no intersectional AV range for the tetragonal arrangement and the octagonal arrangement.

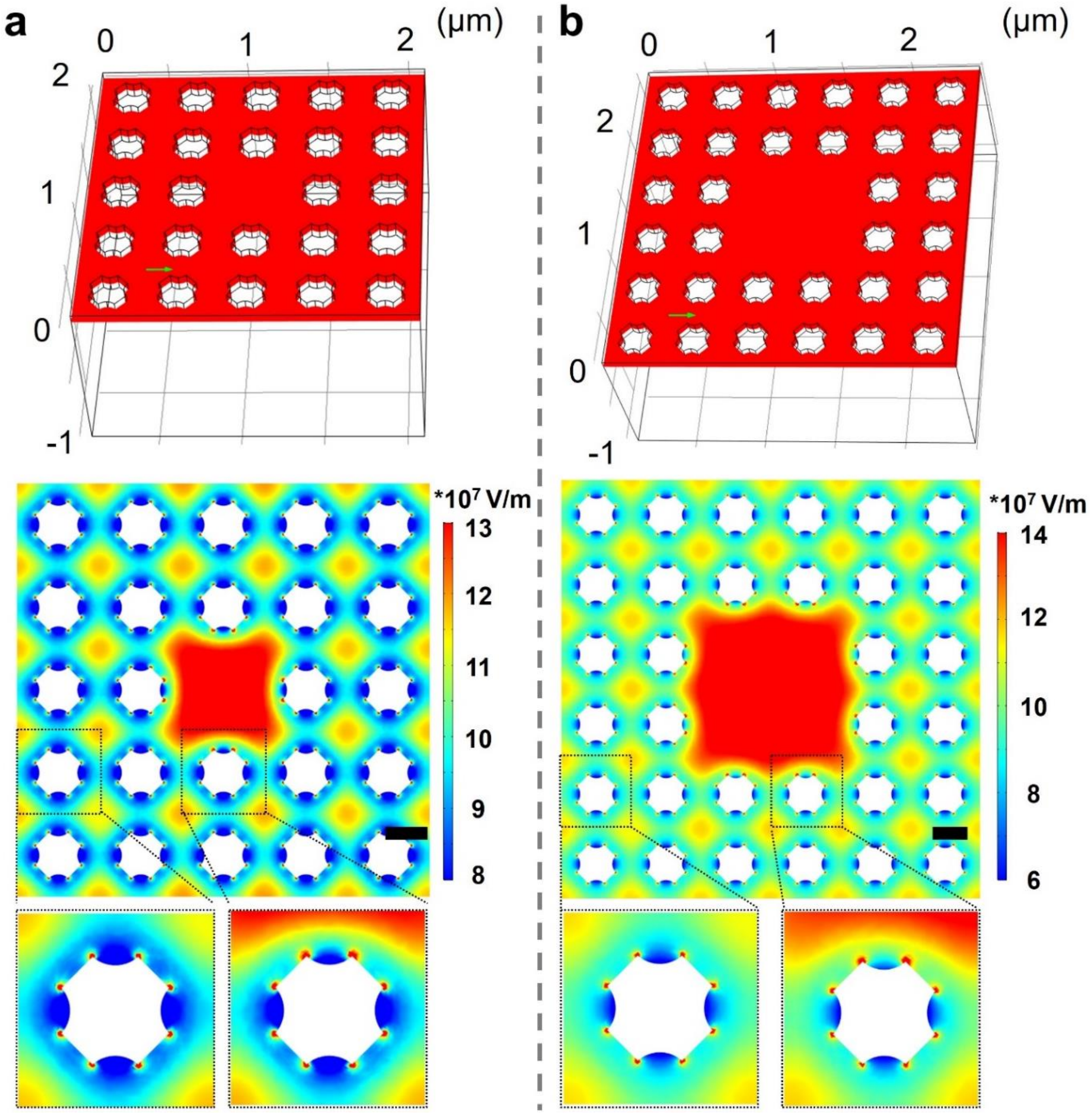

Supplementary Fig. 25 | COMSOL-simulated electric field maps across the near-surface lateral cross sections of surface-patterned aluminium foils. (**a**) Three-dimensional layout and electric field distribution for the surface-patterned aluminium foil with spacing-different tetragonal arrangements. The nanoconcave array is based on the configuration in Supplementary Fig. 22c. (**b**) Three-dimensional layout and electric field distribution for the surface-patterned aluminium foil with a mixture of tetragonal and octagonal arrangements. The nanoconcave array is based on the configuration in Supplementary Fig. 24c. For a clear comparison, the electric field maps in the basic (left, down) and accessory (right, down) arrangements are illustrated with higher magnification. Obviously, the electric field distribution in the accessory arrangement has higher asymmetry than that in the basic arrangement. All scale bars: 200 nm.

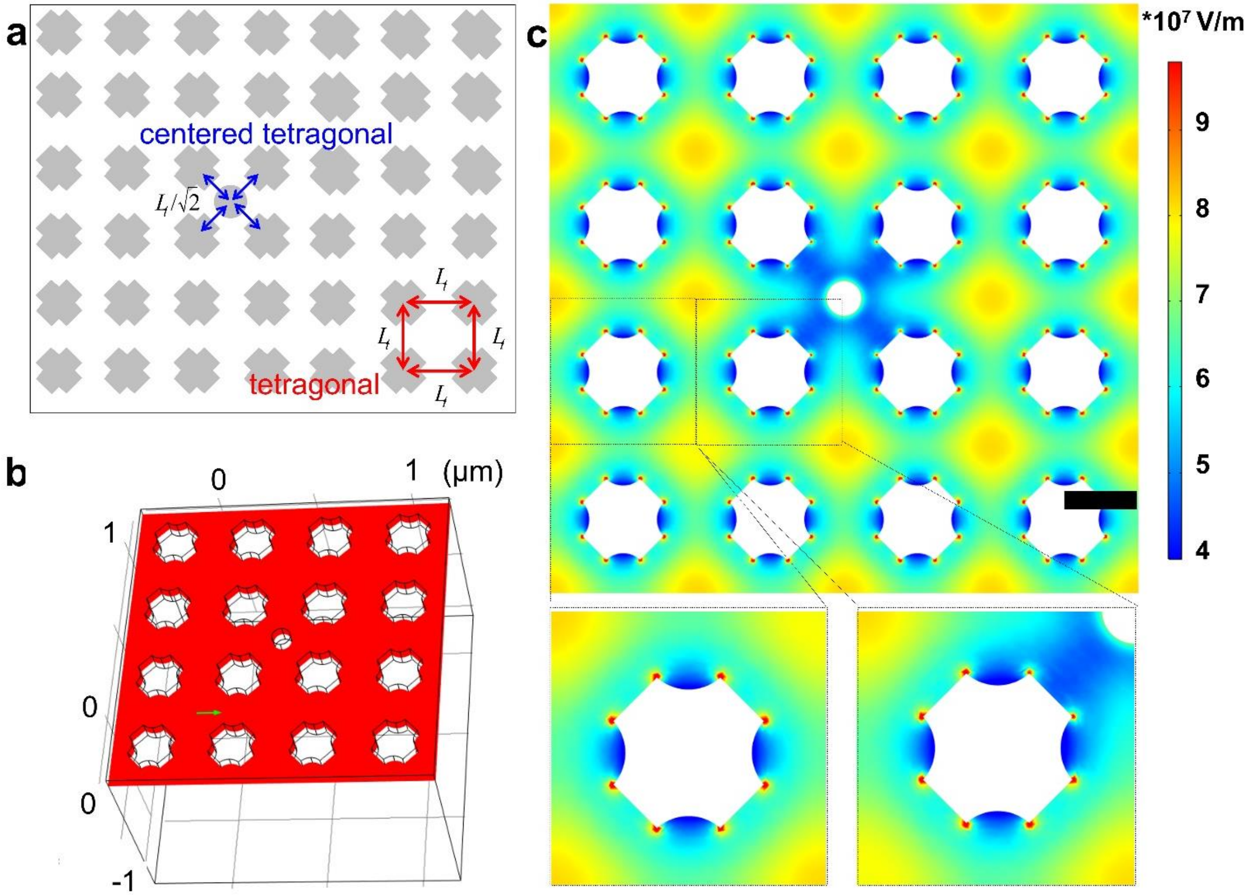

Supplementary Fig. 26 | COMSOL simulation for electric field map of surface-patterned aluminium foil with nanoconcaves of the tetragonal and centered tetragonal arrangements. (**a**) Schematic illustration and (**b**) three-dimensional layout of surface-patterned aluminium foil in COMSOL simulation. For obtaining nanoconcaves of mixture arrangement, we used a tetragonal imprint stamp to equip the aluminium surface with a tetragonal array of nanoconcaves, followed by etching one more nanoconcave at the fourfold junction site of existing nanoconcaves with Zeiss FIB-SEMs system. (**c**) COMSOL-simulated electric field maps across the near-surface lateral cross section. Scale bar: 200 nm.

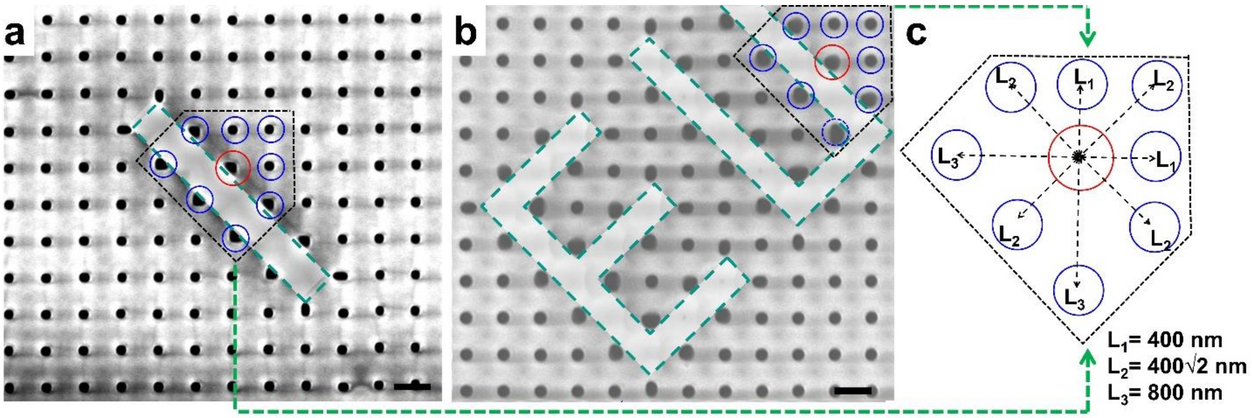

Supplementary Fig. 27 | Spatial configuration of triangular pores in mixture arrangements. (**a**,**b**) SEM images of mixture-arrangement templates. The SEM images in (**a**) and (**b**) are the same as those in Fig. 4d,f, respectively. (**c**) Schematic illustration for the spatial configuration of the triangular pore marked by a red circle. The triangular pores in (**a**) and (**b**) have the same spatial configuration.

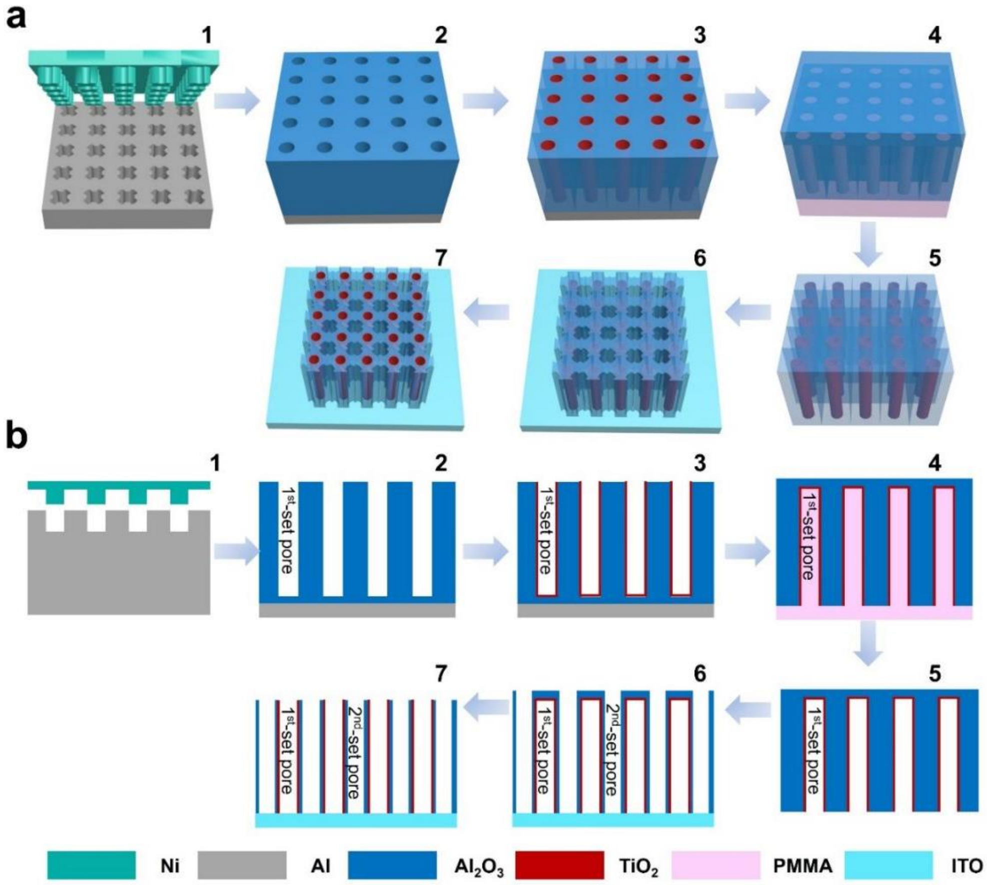

Supplementary Fig. 28 | Realization of the 2nd-set and two-set pores in AAO template. Schematic illustration of the fabrication process in (**a**) three-dimensional view and (**b**) two-dimensional cross-sectional view. The fabrication process includes seven procedures: (1) imprinting an aluminium foil; (2) anodizing the imprinted area to obtain the 1st-set pores; (3) coating a TiO_2 layer along the walls of the 1st-set pores to protect the 1st-set pores in the following etching procedures; (4) dripping PMMA over the as-anodized area to serve as a supporting scaffold to prevent the AAO template from cracking, followed by removing the unanodized aluminium; (5) dissolving PMMA by acetone; (6) opening the 2nd-set circular pores by using 0.1 M NaOH solutions, followed by reshaping the 2nd-set pores from a circular shape to a 4-edged cross shape because of uneven etching rates of pore walls in H_3PO_4 solutions, and then transferring the AAO template on an ITO substrate; (7) opening the 1st-set pores by ion-milling. Here the schematic illustration takes a tetragonal pore-arrangement template as an example, and the fabrication processes for templates with other pore arrangements (*e.g.*, the hexagonal and mixture arrangements) are similar. The templates in Fig. 5b_1-b_4 and Fig. 5d_1-d_4 correspond to the 2nd-set pore arrays without removing the barrier layer of the 1st-set pores (procedure 6). And the templates in Figs. 5a_ib_j and 4c_md_n correspond to the two-set pore arrays after ion-milling off the barrier layer of the 1st-set pores (procedure 7). Note that, the size and shape of the 2nd-set pores can be readily tuned by controlling etching time in NaOH and H_3PO_4 solutions, as evidenced by Fig. 5.

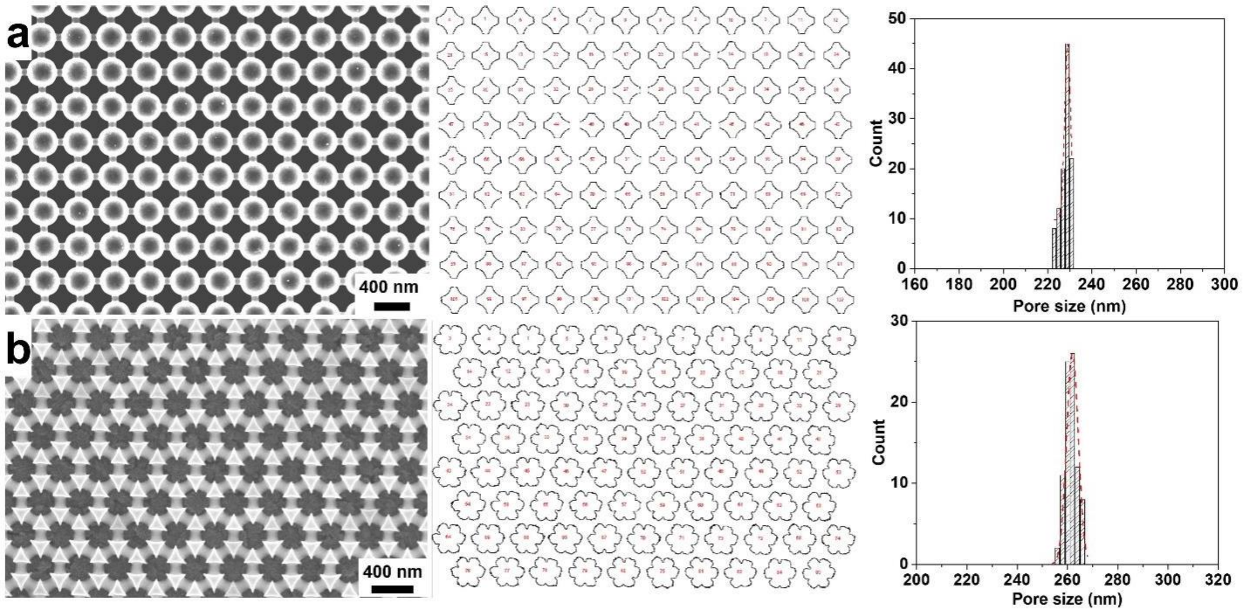

Supplementary Fig. 29 | Size distribution of the 2nd-set pores in AAO templates. The 2nd-set pores are arrayed in the (**a**) tetragonal and (**b**) trigonal arrangements.

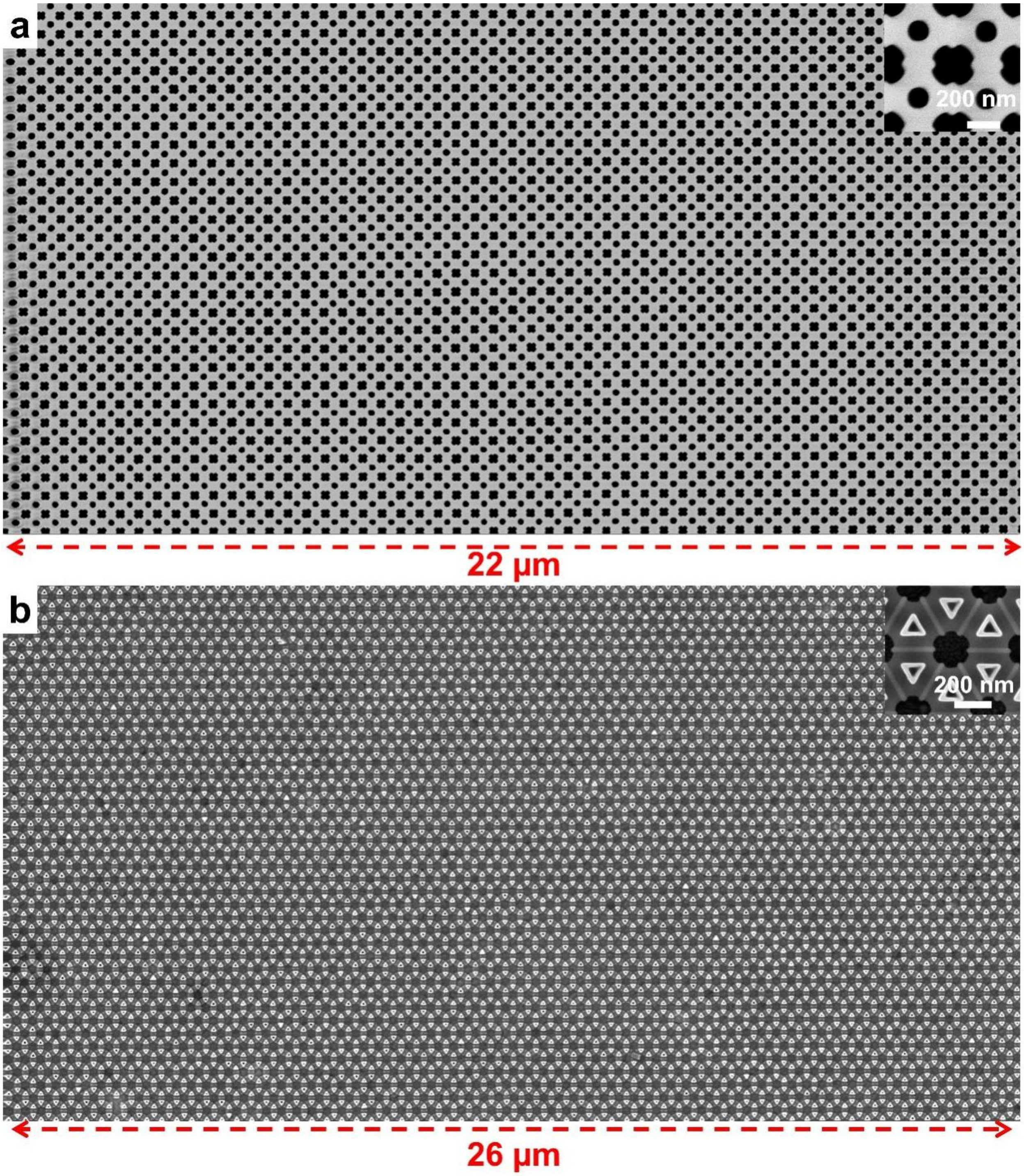

Supplementary Fig. 30 | Large-area SEM images of AAO templates with two sets of shape-different pores: (**a**) the same type of template as the one in Fig. 5a_1b_3; (**b**) the same type of template as the one in Fig. 5c_1d_3. Insets: the corresponding SEM images with higher magnification.

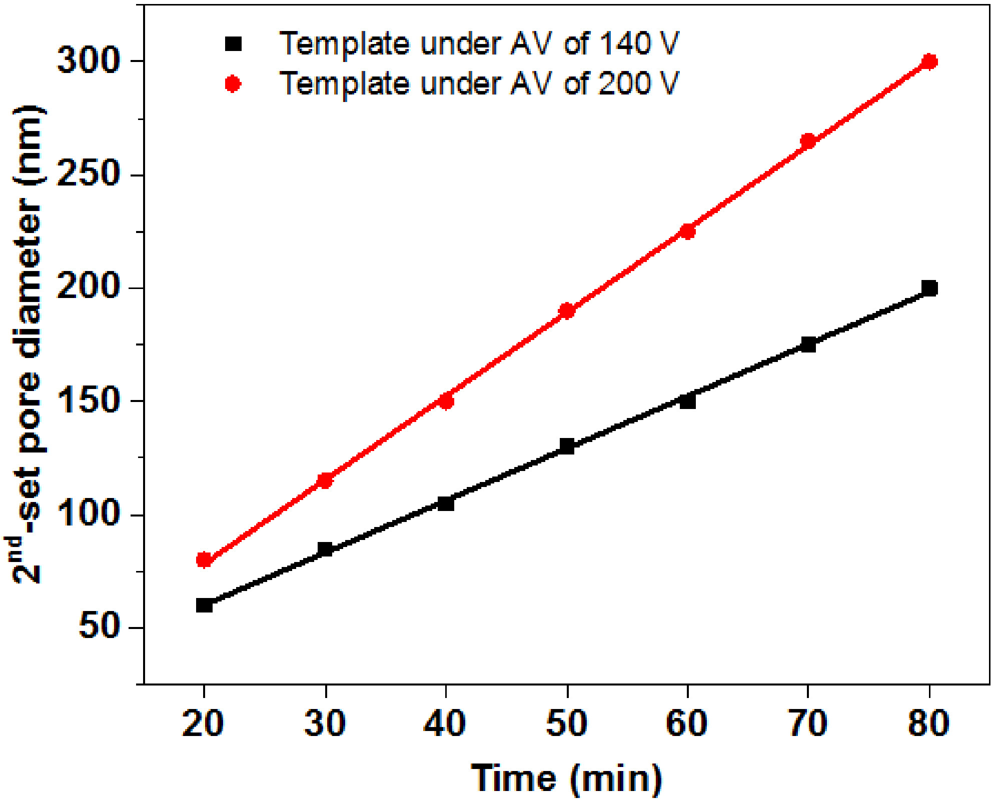

Supplementary Fig. 31 | Dependence of the 2nd-set pore diameter on etching time in NaOH solutions. The templates were anodized under AVs of 140 V (black squares) and 200 V (red circles), respectively. According to the linear fitting analysis, the corresponding etching rates of the 2nd-set pores for two templates are about 2.3 and 3.7 nm/min, respectively.

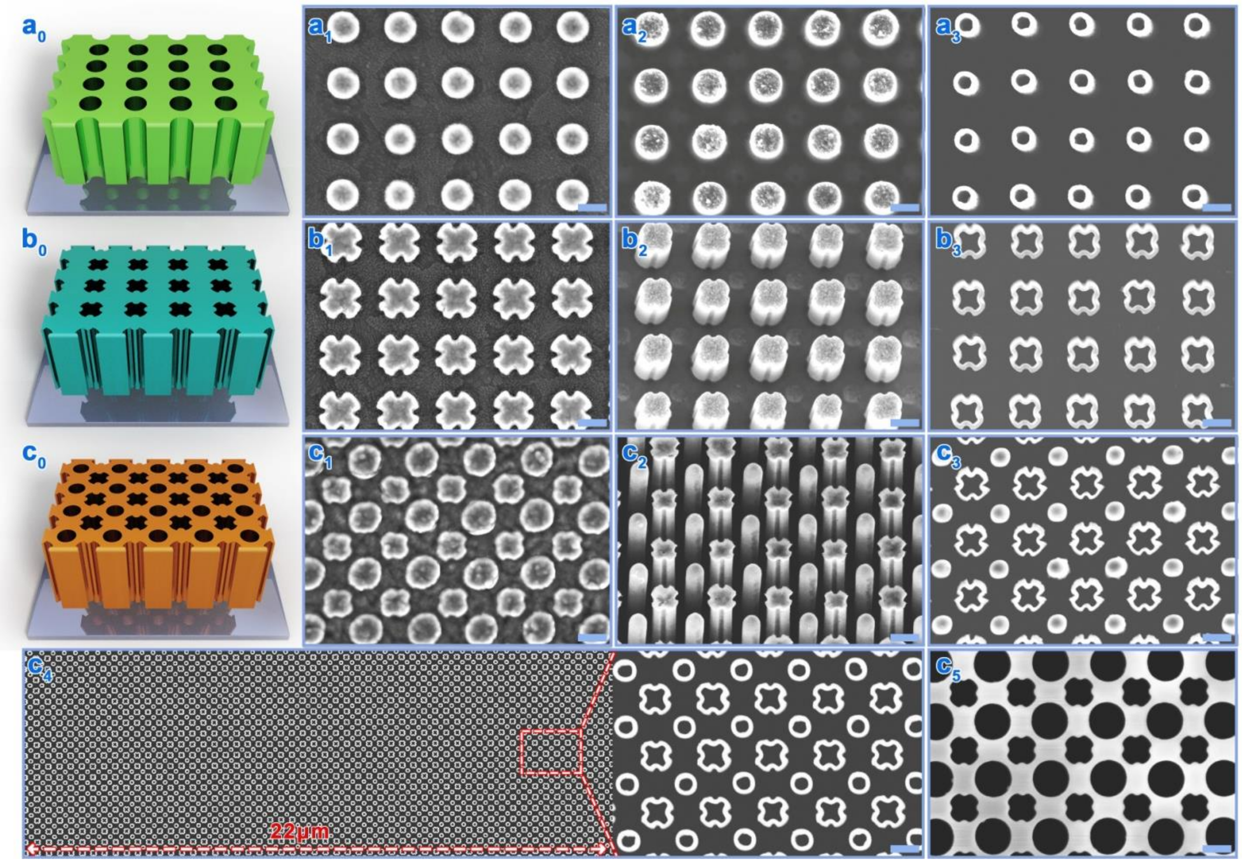

Supplementary Fig. 32 | Nanostructure arrays replicated from designable templates. (**a**$_0$) Schematic of the template with the 1st-set circular pores and SEM images of the resultant nanostructure arrays: (**a**$_1$) Au nanoparticles, (**a**$_2$) Ni nanowires, and (**a**$_3$) TiO_2 nanotubes. The nanostructures in (**a**$_1$-**a**$_3$) feature circular shape, inherited from the 1st-set pores (**a**$_0$). (**b**$_0$) Schematic of the template with the 2nd-set 4-edged cross-shaped pores and SEM images of the resultant nanostructure arrays: (**b**$_1$) Au nanoparticles, (**b**$_2$) Au nanowires, and (**b**$_3$) SnO_2 nanotubes. The nanostructures in (**5b**$_1$-**b**$_3$) replicate the 4-edged cross-shape of the 2nd-set pores (**b**$_0$). (**c**$_0$) Schematic of the template with the two-set pores and SEM images of the resultant nanostructure combinations: (**c**$_1$) Au-nanoparticles/Au-nanoparticles, (**c**$_2$) Pt-nanowires/Au-nanowires, (**c**$_3$) Pt-nanowires/SnO_2-nanotubes, (c$_4$) TiO_2-nanotubes/SnO_2-nanotubes, (**c**$_5$) Ni dual-pore nanomesh. The nanostructures in (**c**$_1$-**c**$_5$) combine the shapes of both pores (**c**$_0$). Scale bars: 200 nm.

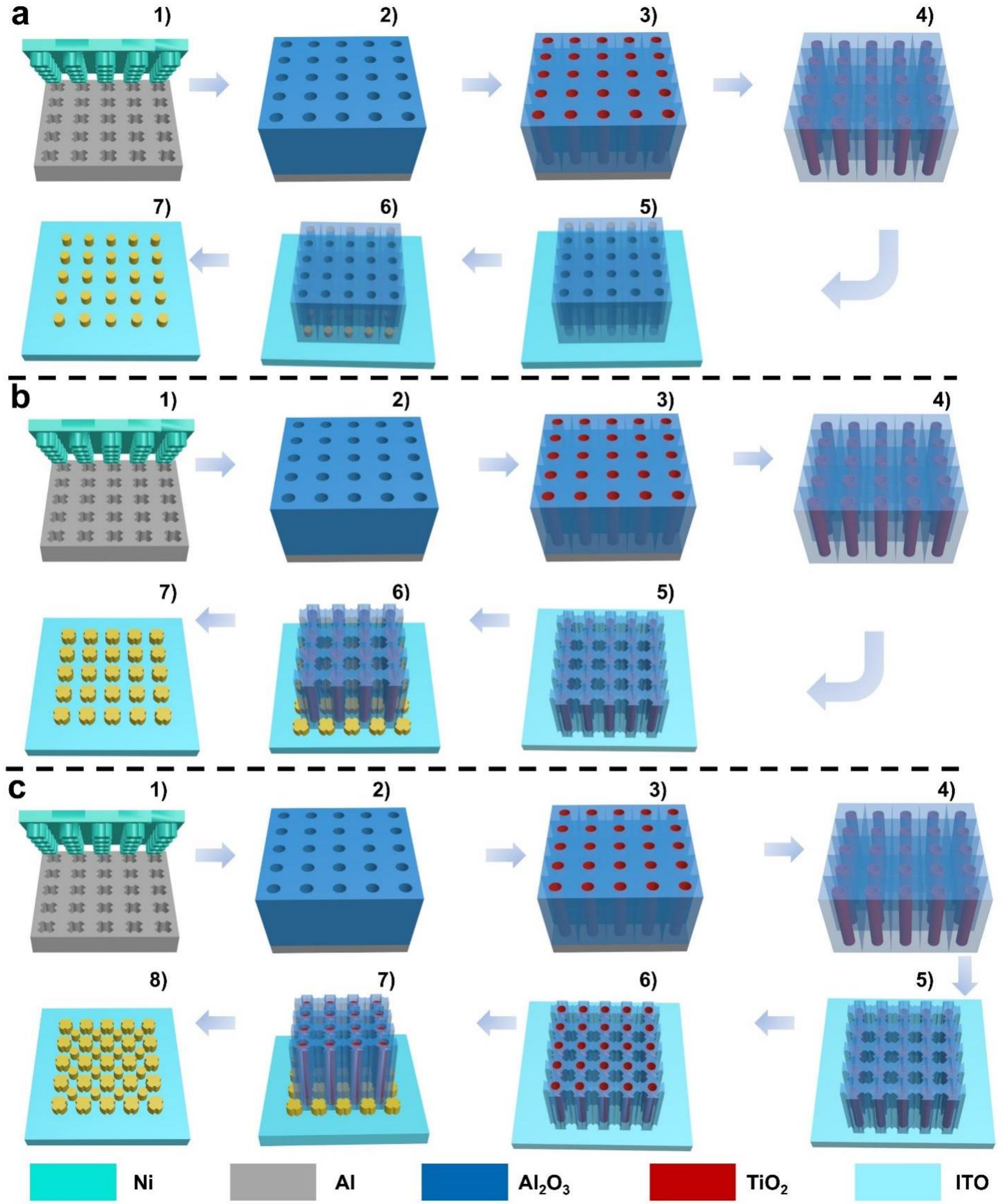

Supplementary Fig. 33 | Fabrication of zero-dimensional nanoparticle arrays using AAO template. Schematic illustration of the fabrication process of Au nanoparticles replicating the shapes of the (**a**) 1st-set, (**b**) 2nd-set, and (**c**) two-set pores, respectively.

To construct tetragonally arranged Au nanoparticles, the lab-made Ni imprint stamp with a tetragonal array of four-leaf clover-like nanopillars anchored on its surface was exploited for obtaining four-leaf clover-like nanoconcaves on aluminium foil surface at a constant pressure of 10 kN cm^{-2} for 3min (Step 1). The imprinted area with periodic nanoconcaves was anodized at an AV of 200 V, achieving a tetragonal array of circular pores (Step 2). After anodization, a 10-nm-thick TiO_2 layer was coated along the pore walls by ALD, followed by ion-milling off the TiO_2 layer at the top surface (Step 3). Then PMMA solution was dripped onto the anodized area of the aluminium foil and got dried naturally. With the dried PMMA as a supporting scaffold, the unanodized aluminium material was wet-chemically etched, finally achieving a thin template with circular pores after dissolving PMMA by acetone (Step 4).

In Supplementary Fig. 33a, the AAO template was then transferred onto an ITO substrate, followed by ion-milling off the barrier layer at the bottom of the 1st-set pores (Step 5). After completely opening the 1st-pores, Au material was evaporated onto the AAO template by PVD (Step 6). After peeling off the AAO template, an array of Au nanoparticles was obtained, which inherited the circular shape of the 1st-set pores (Step 7), as illustrated in Supplementary Fig. $32a_1$.

In Supplementary Fig. 33b, the AAO template was then immersed into 0.1 M NaOH solutions for 30 min, giving rise to the 2nd-set pores with a circular shape located at the junction sites of neighboring four 1st-set pores. The 2nd-set pores were further reshaped into a 4-edged cross shape in H_3PO_4 solutions. Afterwards, the AAO template owning the opened 2nd-set pores and the blocked 1st-set pores (without removing the barrier layer) was transferred onto an ITO substrate (Step 5). Finally, an array of Au nanoparticles replicating the 2nd-set pore shape was obtained after evaporating Au material (Step 6) and removing the template (Step 7), as illustrated in Supplementary Fig. $32b_1$.

In Supplementary Fig. 33c, after opening and reshaping the 2nd-set pores (Step 5), the barrier layer of the 1st-set pores was also ion-milled off, leading to an AAO template including two sets of completely opened pores (Step 6). In the same way, an array combining two sets of Au nanoparticles was obtained (Steps 7 and 8), as illustrated in Supplementary Fig. $32c_1$. The nanoparticle heights of all samples can be easily controlled by the PVD parameters such as evaporation rate and time.

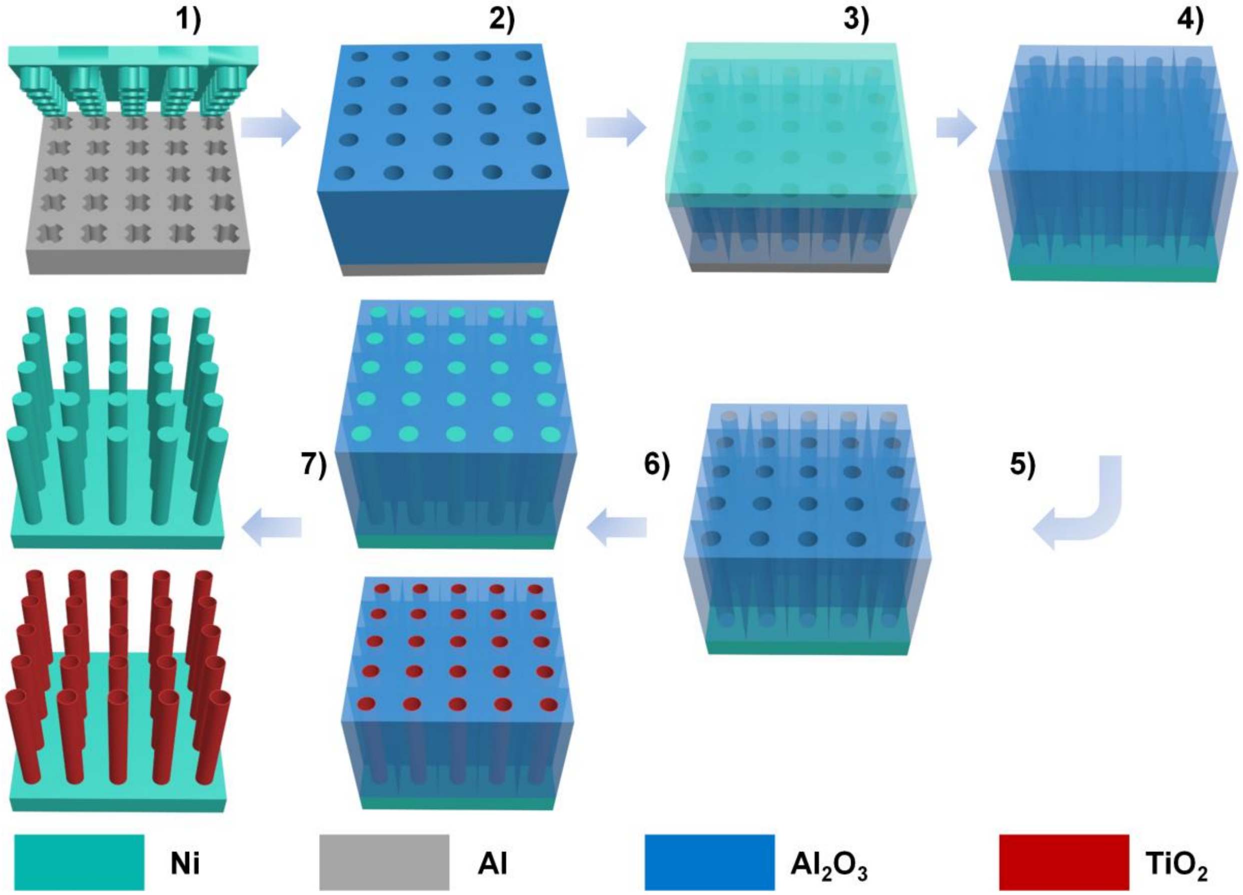

Supplementary Fig. 34 | Fabrication of one-dimensional nanostructure arrays using the 1st-set pores of AAO template.

After obtaining the 1st-set pores by anodizing surface-patterned aluminium foils according to the strategy introduced in Supplementary Fig. 33 (Steps 1 and 2), a conductive layer (5-nm-thick Ti and 20-nm-thick Au) was evaporated onto the top surface, followed by electrodeposition of a thick Ni layer (Step 3). Afterwards, the unanodized aluminium was wet-chemically etched (Step 4), and the exposed barrier layer of the 1st-set pores was ion-milled off (Step 5). Using the 1st-set pores as template, Ni nanowires were electrodeposited into the pores. Likewise, TiO_2 nanotubes can be produced along the pore walls by ALD, followed by ion-milling off the TiO_2 layer on the surface (Step 6). Finally, the AAO template was dissolved by NaOH solutions (Step 7), realizing arrays of Ni nanowires (Supplementary Fig. 32a$_2$) and TiO_2 nanotubes (Supplementary Fig. 32a$_3$) that replicated the circular shape of the 1st-set pores.

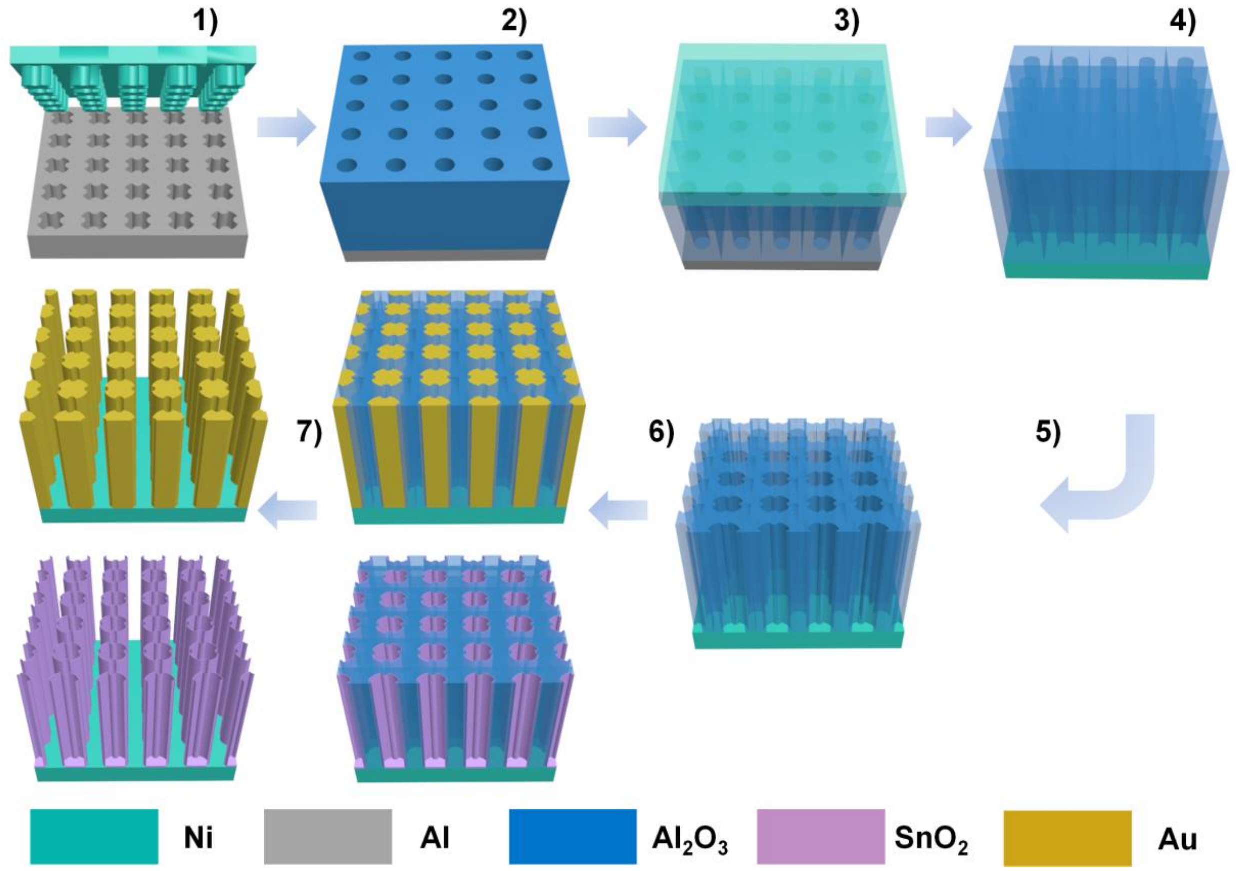

Supplementary Fig. 35 | Fabrication of one-dimensional nanostructure arrays using the 2nd-set pores of AAO template.

After anodizing surface-patterned aluminium foils following the approach introduced in Supplementary Fig. 33 (Steps 1 and 2), electrodepositing a thick Ni substrate with PVD-produced Ti/Au film as the working electrode (Step 3), and removing the unanodized aluminium (Step 4) according to the strategy used in Supplementary Fig. 34, the 2nd-set pores were opened in NaOH solutions and then reshaped in H_3PO_4 solutions (Step 5). After that, Au nanowires (or SnO_2 nanotubes) were grown into the 2nd-set pores by electrodeposition (or ALD), respectively (Step 6). After etching the AAO template in NaOH solutions, Au nanowires (or SnO_2 nanotubes) were constructed on the Ni substrate (Step 7), which were characterized by the 4-edged cross shape of the 2nd-set pores (Supplementary Fig. 32b2,b3).

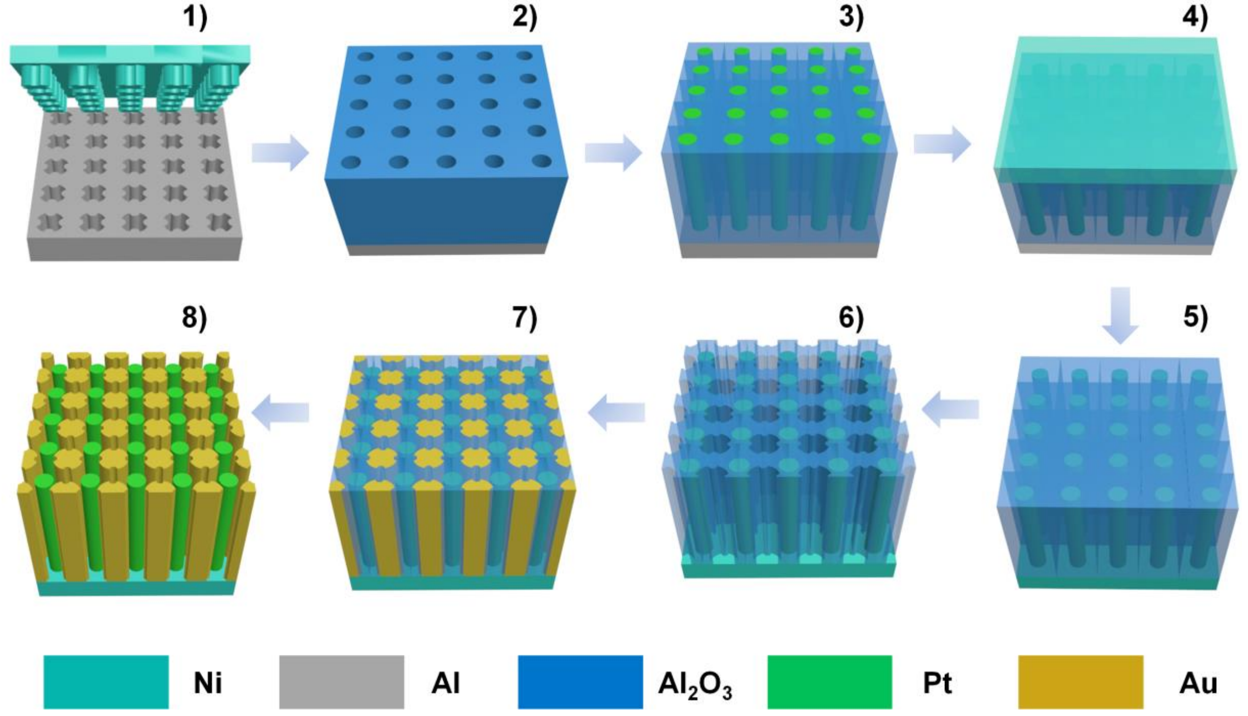

Supplementary Fig. 36 | Fabrication of nanowires/nanowires array using AAO template with pore combination.

After obtaining the 1st-set pores (Steps 1 and 2) according to the strategy stated in Supplementary Fig. 33, ALD of Pt material was performed to form Pt nanowires in the 1st-set pores (Step 3). Then, a thick Ni substrate was electrodeposited to support the AAO template (Step 4). The unanodized aluminium on the backside was then wet-chemically removed (Step 5). Upon the exposed AAO template, the 2nd-set pores were opened in NaOH solutions and then reshaped in H_3PO_4 solutions (Step 6). After exposing the conductive Ni substrate, electrodeposition was conducted to form Au nanowires in the 2nd-set pores (Step 7). Finally, the AAO template was wet-chemically etched, leading to a Pt-nanowires/Au-nanowires array on the Ni substrate (Step 8). As shown in Supplementary Fig. $32c_2$, the fabricated Pt-nanowires/Au-nanowires replicated the shapes of two sets of pores, respectively.

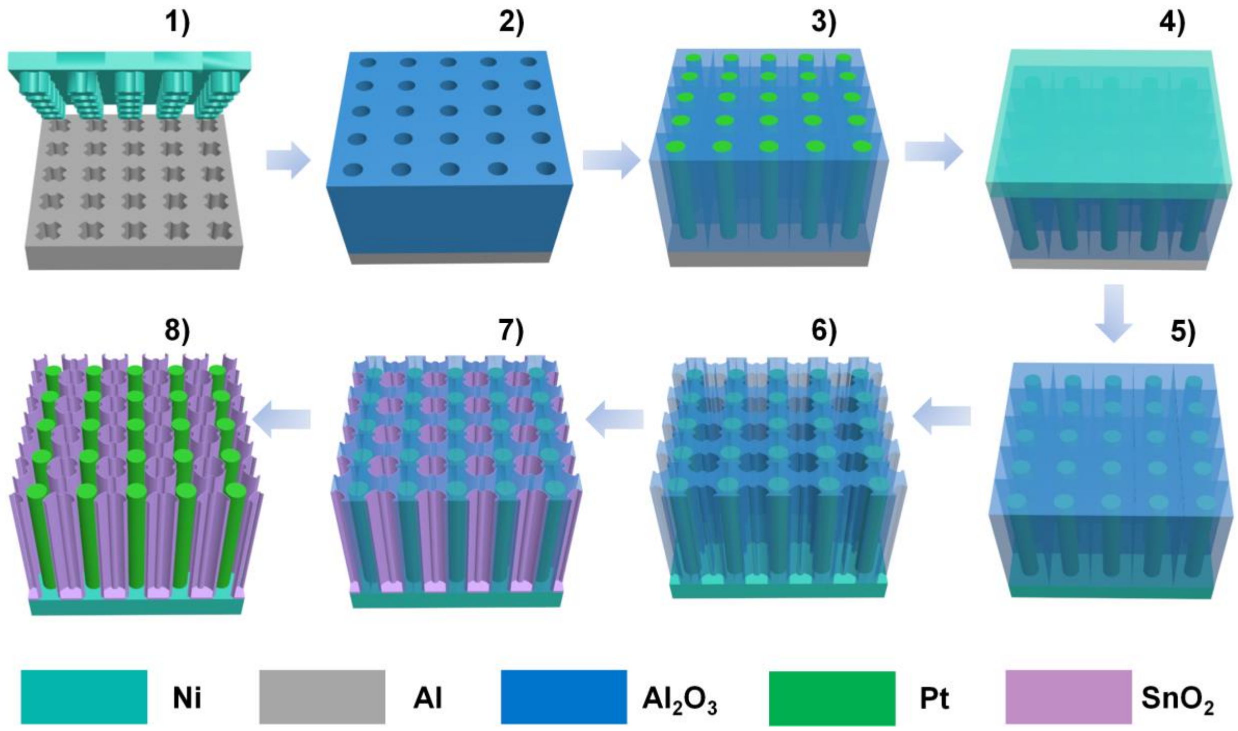

Supplementary Fig. 37 | Fabrication of nanowires/nanotubes array using AAO template with pore combination.

As for the Pt-nanowires/SnO_2-nanotubes array, Pt nanowires were formed in the 1st-set pores, and a thick Ni layer was then electrodeposited to support the AAO template, following the methods for the fabrication of Pt-nanowires/Au-nanowires array in Supplementary Fig. 36 (Steps 1 to 4). After wet-chemically removing the unanodized aluminium on the backside and etching the 2nd-set pores sequentially in NaOH solutions and H_3PO_4 solutions (Steps 5 and 6), a 20-nm-thick SnO_2 layer was coated along the 2nd-set pores by ALD, followed by ion-milling off the SnO_2 layer on the surface (Step 7). Finally, a Pt-nanowires/SnO_2-nanotubes array was constructed on the Ni substrate after removing the AAO template in NaOH solutions (Step 8). As shown in Supplementary Fig. 32c_3, the fabricated Pt-nanowires/SnO_2-nanotubes replicated the shapes of two sets of pores, respectively.

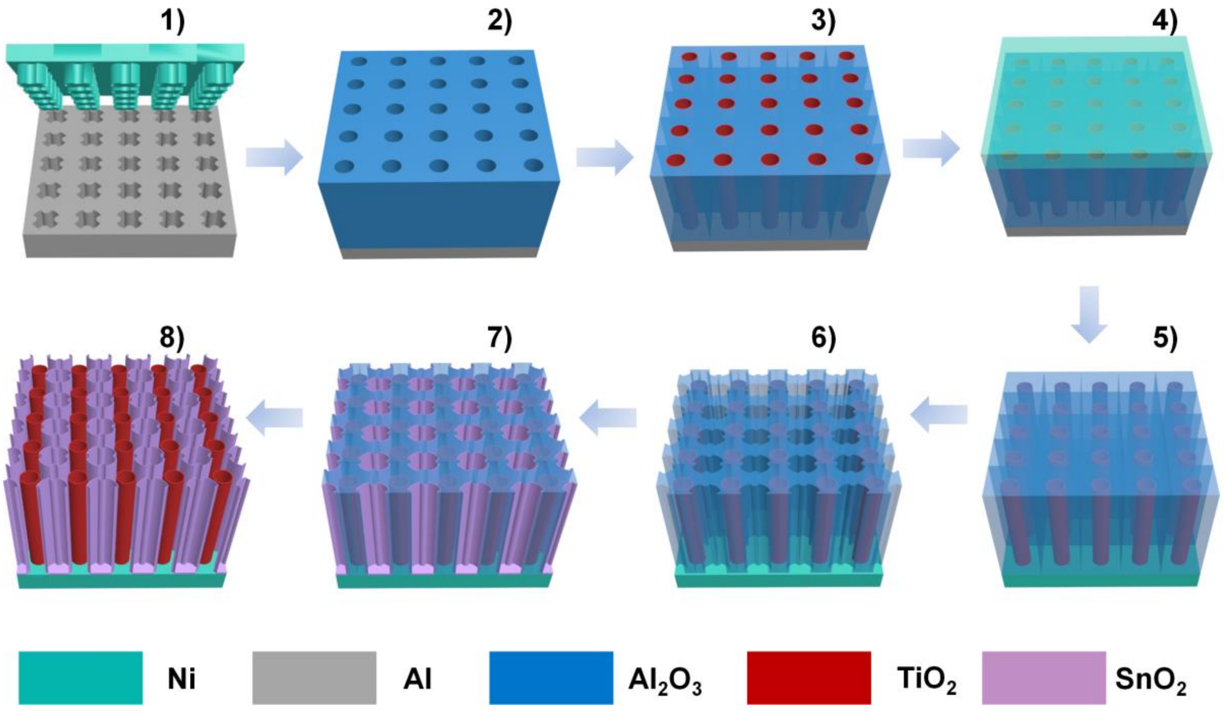

Supplementary Fig. 38 | Fabrication of nanotubes/nanotubes array using AAO template with pore combination.

Regarding the fabrication of TiO_2-nanotubes/SnO_2-nanotubes arrays, the 1st-set pores were obtained following the strategy introduced in Supplementary Fig. 33 (Steps 1 and 2). Then, a 20-nm-thick TiO_2 layer was coated over the walls of the 1st-set pores, followed by ion-milling off the TiO_2 layer on the top surface (Step 3). Afterwards, a metallic layer (5-nm-thick Ti and 20-nm-thick Au) was evaporated onto the top surface of the aluminium foil by PVD. With this metallic layer as a conductive electrode, Ni electrodeposition was conducted to form a thick substrate (Step 4). The unanodized aluminium was removed, and the 2nd-set pores were then opened in the etching solutions (Steps 5 and 6). ALD was performed again to coat another SnO_2 layer along the walls of the 2nd-set pores, followed by ion-milling off the SnO_2 on the surface (Step 7). After wet-chemically dissolving the AAO template (Step 8), an array of TiO_2-nanotubes/SnO_2-nanotubes was constructed on the Ni substrate. As illustrated in Supplementary Fig. 32c$_4$, the fabricated TiO_2-nanotubes/SnO_2-nanotubes replicated the shapes of two sets of pores, respectively.

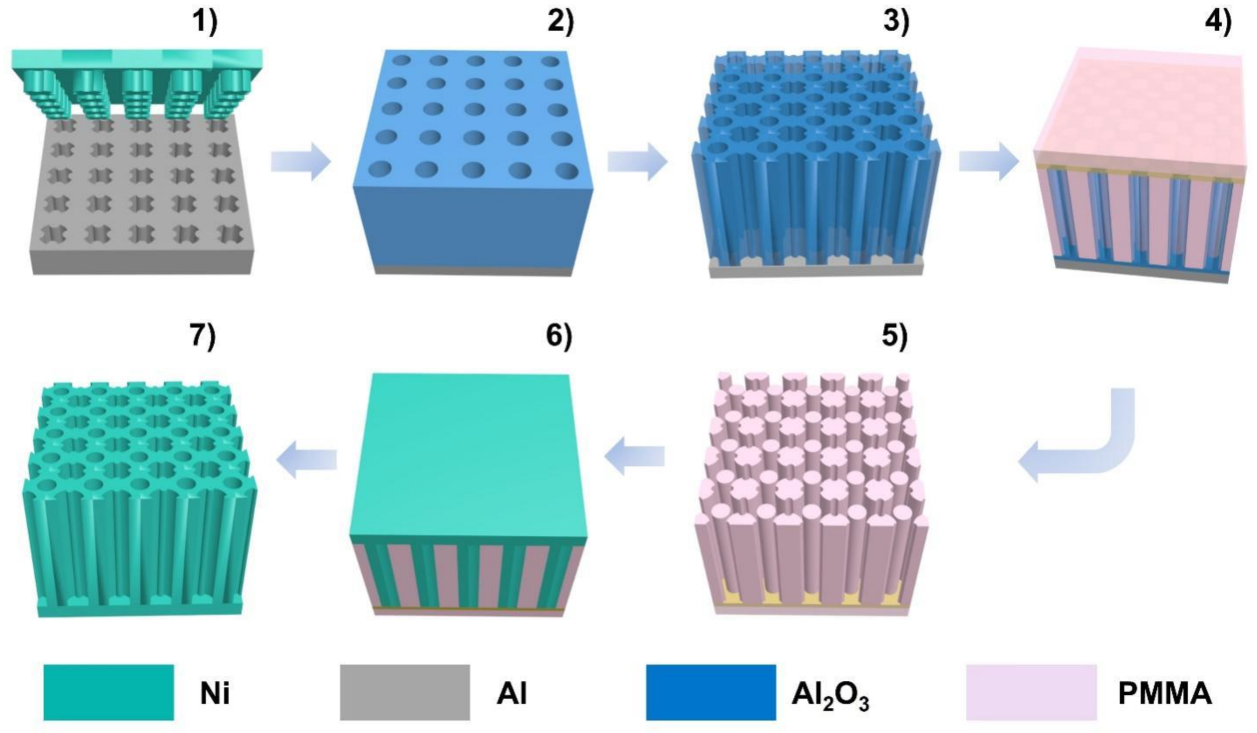

Supplementary Fig. 39 | Fabrication of Ni nanomesh using AAO template with pore combination.

After performing anodic anodization over surface-imprinted aluminium foils according to the approach introduced in Supplementary Fig. 33 (Steps 1 and 2), the barriers of the 2nd-set pores were thinned down by ion-milling, followed by pore-opening in NaOH solutions and then pore-reshaping in H_3PO_4 solutions (Step 3). Upon the as-anodized area, a 20-nm-thick Au layer was evaporated by PVD at a low evaporation rate of 0.01 nm/min, followed by coating PMMA solution (Step 4). After naturally drying PMMA, the unanodized aluminium and the AAO template were wet-chemically removed (Step 5). Afterwards, Ni deposition was performed with the exposed Au layer as working electrode (Step 6). Finally, PMMA was dissolved by acetone, leading to a Ni nanomesh with two sets of pores (Step 7), as illustrated in Supplementary Fig. 32c_5.

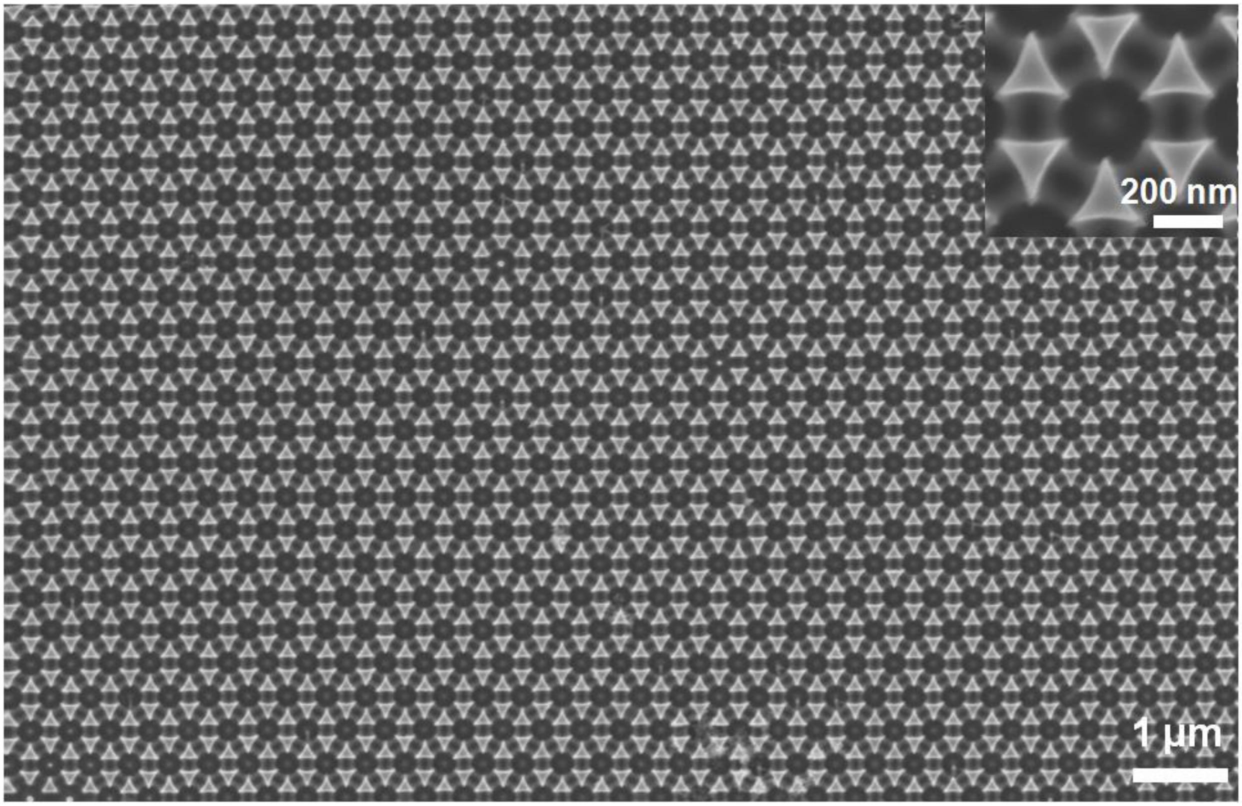

Supplementary Fig. 40 | Large-area hexagonal array of Ag nanoparticles with internally-bent triangular shape. This SEM image is obtained from the sample S3 (shown in Fig. 5a), which is characterized by the best device performance. The Ag nanoparticles possess reliable structural uniformity, which is in favor of yielding homogeneous and reproducible SERS signals over large areas.

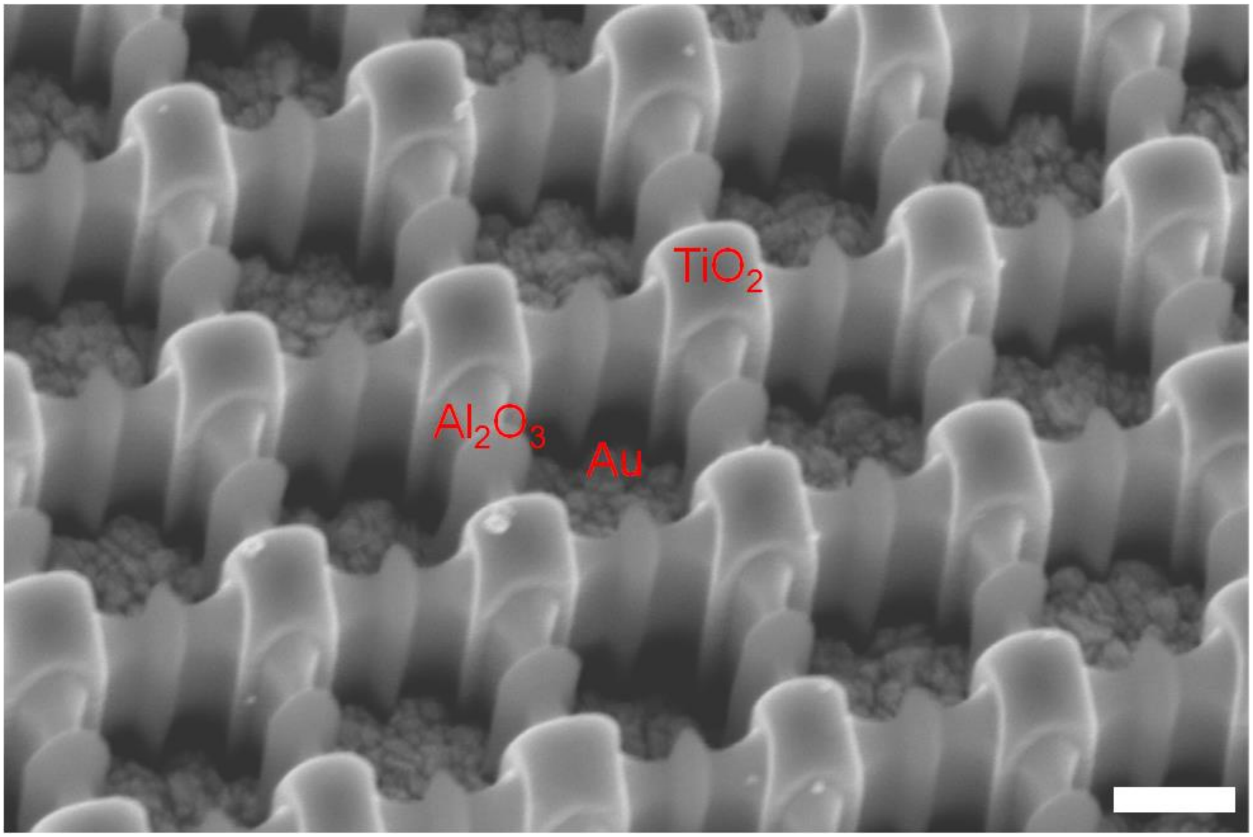

Supplementary Fig. 41 | TiO_2-nanotubes/Au-nanowires combination in AAO template. Scale bar: 200 nm.

Supplementary References:

1. Vrublevsky, I., Parkoun, V., Sokol, V., Schreckenbach, J. & Marx, G. The study of the volume expansion of aluminum during porous oxide formation at galvanostatic regime. *Appl. Surf. Sci.* **222**, 215-225 (2004).
2. Lee, W. & Park, S.-J. Porous anodic aluminum oxide: anodization and templated synthesis of functional nanostructures. *Chem. Rev.* **114**, 7487-7556 (2014).
3. Nielsch, K., Choi, J., Schwirn, K., Wehrspohn, R. B. & Gösele, U. Self-ordering regimes of porous alumina: the 10 porosity rule. *Nano Lett.* **2**, 677-680 (2002).

4 Smith, J. T., Hang, Q., Franklin, A. D., Janes, D. B. & Sands, T. D. Highly ordered diamond and hybrid triangle-diamond patterns in porous anodic alumina thin films. *Appl. Phys. Lett.* **93**, 043108 (2008).
